# Embodied Digital Technologies: First Insights in the Social and Legal Perception of Robots and Users of Prostheses

**DOI:** 10.3389/frobt.2022.787970

**Published:** 2022-04-11

**Authors:** Sarah Mandl, Maximilian Bretschneider, Stefanie Meyer, Dagmar Gesmann-Nuissl, Frank Asbrock, Bertolt Meyer, Anja Strobel

**Affiliations:** ^1^ Personality Psychology and Assessment, Institute of Psychology, Chemnitz University of Technology, Chemnitz, Germany; ^2^ Social Psychology, Institute of Psychology, Chemnitz University of Technology, Chemnitz, Germany; ^3^ Work, Organizational, and Economic Psychology, Institute of Psychology, Chemnitz University of Technology, Chemnitz, Germany; ^4^ Private Law and Intellectual Property Rights, Faculty of Economics and Business Administration, Chemnitz University of Technology, Chemnitz, Germany

**Keywords:** legal perception, social perception, robots, bionics, prosthetics, anthropomorphism, stereotypes

## Abstract

New bionic technologies and robots are becoming increasingly common in workspaces and private spheres. It is thus crucial to understand concerns regarding their use in social and legal terms and the qualities they should possess to be accepted as ‘co-workers’. Previous research in these areas used the Stereotype Content Model to investigate, for example, attributions of Warmth and Competence towards people who use bionic prostheses, cyborgs, and robots. In the present study, we propose to differentiate the Warmth dimension into the dimensions of Sociability and Morality to gain deeper insight into how people with or without bionic prostheses are perceived. In addition, we extend our research to the perception of robots. Since legal aspects need to be considered if robots are expected to be ‘co-workers’, for the first time, we also evaluated current perceptions of robots in terms of legal aspects. We conducted two studies: In Study 1, participants rated visual stimuli of individuals with or without disabilities and low- or high-tech prostheses, and robots of different levels of Anthropomorphism in terms of perceived Competence, Sociability, and Morality. In Study 2, participants rated robots of different levels of Anthropomorphism in terms of perceived Competence, Sociability, and Morality, and additionally, Legal Personality, and Decision-Making Authority. We also controlled for participants’ personality. Results showed that attributions of Competence and Morality varied as a function of the technical sophistication of the prostheses. For robots, Competence attributions were negatively related to Anthropomorphism. Perception of Sociability, Morality, Legal Personality, and Decision-Making Authority varied as functions of Anthropomorphism. Overall, this study contributes to technological design, which aims to ensure high acceptance and minimal undesirable side effects, both with regard to the application of bionic instruments and robotics. Additionally, first insights into whether more anthropomorphized robots will need to be considered differently in terms of legal practice are given.

## Introduction to Social Perception of Embodied Digital Technologies

Social Perception influences social interaction in societies (Cuddy et al., 2008). Hybrid societies include human actors and Embodied Digital Technologies (EDTs). These societies are not a thing of the distant future anymore. Bionics users are becoming common, as are robots in workspace settings. The perception of these new kinds of actors, and their subsequent roles and acceptance within a society, is the focus of current studies.

The Stereotype Content Model (SCM; Fiske et al., 2002) identifies Warmth and Competence as the two major dimensions of Social Perception and stereotyping. Social groups can be categorized on these dimensions, for example, housewives are perceived as warm, but incompetent, while feminists are perceived as cold, but competent (Fiske et al., 2002). In the context of hybrid societies, Meyer and Asbrock (2018) showed that bionic prostheses affect attributions of Warmth and Competence towards their users. When using bionic prostheses, the perception of people with disabilities changes. They regain perceived Competence while maintaining the warmer perception of people with disabilities without bionic prostheses. Users of bionic technologies who aim at augmenting their capabilities rather than at restoring functionalities are sometimes described as cyborgs. Cyborgs are perceived as colder and more competent than their purely human counterparts (Meyer & Asbrock, 2018). In the current manuscript, our aim is to not only replicate these findings, but also to extend them to the perception of robots. We see the step from cyborgs to robots as an abstraction. Where cyborgs are inherently human, robots lose these qualities. The absence of these qualities affects their perception in terms of Warmth and Competence, and subsequently, their acceptance. This is important mainly within the context of workspaces. The number of robots is steadily increasing, e.g., from 18,800 in 2010 to 30,000 in 2020 for Germany (*Statistisches Bundesamt*, 2021). Industrial settings were the first areas where robots were utilized to assist and, in some cases, replace human workers (Ben-Ari & Mondada, 2018). Already in the 1980s, robot implementation programs evoked mixed reactions. Low-skill workers reacted more negatively than high-skill workers to robots (Chao & Kozlowski, 1986). Dealing with the (social) issues surrounding human-robot co-working is a crucial success factor for the industry of the future (Demir et al., 2019). Different preferences for working with robots might also be associated with the perception of robots (cf., Davis 1989). Recent ly, Abele et al. (2021) raised critique at the two-dimensional approach of the Stereotype Content Model. This led us to include a third dimension of Social Perception, namely Morality, in the present study. We assume that with the incoming of robots in work- and private spheres, legal adjustments need to be considered, and subsequently accepted by the public. To extend previous findings for users of different prosthesis types (Meyer & Asbrock, 2018) to social robots, two studies addressed three major research questions.

1) How does the public perceive people with disabilities, different types of prostheses as well as different types of robots regarding Competence, Sociability, and Morality?
2) How are personality factors associated with these perceptions?
3) How are different types of robots perceived in terms of Legal Personality and Decision-Making Authority?

## Dimensions of Social Perception

To examine the core dimensions of Social Perception in this context, we employ the Stereotype Content Model (SCM; Fiske et al., 2002) as a theoretical fundament. The Stereotype Content Model originates in social cognition research and has become one of the most prominent theoretical models on Social Perception. It is applied to describe intergroup and interpersonal perception and to understand the perception of consumer brands or artificial intelligence (McKnee et al., 2021). The Stereotype Content Model postulates that all group stereotypes and interpersonal impressions are formed on two fundamental dimensions. These are Warmth (from cold = bad intentions to warm = good intentions) and Competence (from incompetent to competent), resulting in four possible combinations. Persons or groups perceived as warm and incompetent evoke emotions like pity or compassion and elicit active facilitation (help, patronize) as a behavioral correlate. Conversely, people perceived as cold and competent trigger emotions like envy or mistrust that are accompanied by passive harm, for example, ignorance or sabotage (Cuddy et al., 2008). Despite being well established and empirically tested in numerous studies, the Stereotype Content Model has been criticized due to its two-dimensionality (Abele et al., 2021). Regularly, Morality, in the original model included in Warmth (Fiske, 2018), has been brought up as a third factor. This can be achieved by dividing Warmth into two subdimensions of Morality and Sociability (Kervyn et al., 2015). Accordingly, we divided Warmth into Sociability and Morality. Sociability covers how a person or robot is perceived in terms of Likeableness and Warmth. Morality covers how a person or robot is perceived in terms of their intentions to act immoral or moral. The importance of Morality on functioning societies is indisputable (Hannah et al., 2011), an integration of this dimension will therefore enlarge former findings.

### Social Perception of People With Disabilities

Users of bionic prostheses are perceived as more competent than people with disabilities. They maintain perceived Warmth of people with disabilities, that is, they are perceived as warmer than able-bodied individuals (Meyer & Asbrock, 2018). These findings can also be applied to another groundbreaking development: Bionic instruments are used to reestablish or expand the capacities of their users by merging human bodies with technological artifacts to serve a particular purpose. Concerning current augmenting devices, for example, exoskeletons, first results indicate that they are also highly likely to influence the self- and other perception of potential users. Peters and Wischniewski (2019) point out that users may likely perceive themselves as inferior while using an exoskeleton when fulfilling their daily work routines. Wearing an exoskeleton may also lead to stigmatization in the workplace because the user appears dependent on a technological artifact to co-workers (Gilotta et al., 2019; Peters & Wischniewski, 2019). In short, the use of bionic technologies can affect stereotypes towards their users and is also likely to affect interpersonal perceptions on the individual and group level.

### Social Perception of Robots

Bionic devices are highly likely to be prevalent in the near future. Robots have become increasingly common in workspaces and will be expected to act as ‘co-workers’ (Demir et al., 2019). Furthermore, robots will be introduced into private spaces. This presupposes that people want and accept robots in their homes. To be accepted, robots need to possess certain qualities. Whether these qualities can be derived from qualities human beings possess or are attributed is still under debate and needs further consideration. Prior research on the Social Perception of robots using the Robotic Social Attributes Scale (RoSAS; Carpinella et al., 2017) showed that traits similar to the humane perceptions of Warmth and Competence can be ascribed to robots. Both dimensions are also the most important predictors for human preferences concerning different robot behaviors (Scheunemann et al., 2020). Especially in industrial settings, robots are seen as social entities and attributed positive and negative humanlike characteristics (Sauppé and Mutlu, 2015). Perception of robots are also influenced by other aspects such as its design or size (de Visser et al., 2016; Li et al., 2010; Rosenthal-von der Pütten, 2014; Schaefer et al., 2012; von der Pütten and Krämer, 2012). Industrial robots might be exempt from this since their appearance needs to conform to measures of safety and usability (Gesmann-Nuissl, 2019). Whereas anthropomorphic features elicit greater trust (de Visser et al., 2016), too close resemblance to actual human beings might have the opposite effect. The uncanny valley phenomenon (Mori et al., 2012) describes how extraordinarily human-like robots are perceived. Mostly, they are seen as rather eerie and elicit feelings of uneasiness or threat. Therefore, the question of ‘how human-like is too human-like’ is not trivial at all and needs closer inspection. We see this study as groundwork to investigate which qualities a robot should posess to be an accepted partner in a hybrid society. Hence, we focus on the expanded Stereotype Content Model, and investigate whether or not inherently humane properties can be attributed to robots. Against the background of increased research focused on moral machines (Awad et al., 2018; Bigman et al., 2019; Cervantes et al., 2020), we assess Morality to examine if and to what extent people are willing to attribute Morality to robots. Acceptance of robots is not solely explained by the robots’ appearance or behavior, but also by a persons’ preference and the subsequent appliance of it. The Technology Acceptance Model (TAM; Davis, 1989) is a theoretical model of how characteristics of computer-based information systems influence user acceptance and subsequent use of these systems. The model considers external variables, such as perceived usefulness and perceived ease of use which influence the attitude towards and behavioral intention to and actual use of a system. It points out that both usefulness and ease of use is founded within the user (Davis, 1989). This implies that personality factors might be associated with how well a system, in our case a robot, is integrated into workspaces and subsequently into society, but necessarily also in the legal system.

### Individual Differences in Social Perception

We considered a broad range of personality variables associated with the perception of others. Since we are assessing the perceived Morality of others, we considered personality factors which were shown as core variables to be associated with moral behavior (Strobel et al., 2017). Affinity for Technology Interaction (ATI) is the tendency to actively engage in intensive technology interaction (Franke et al., 2019). People who show positive interest in technical gadgets are more likely to interact with and accept robots (Heerink, 2011; de Graaf & Ben Allouch, 2013). Technological affinity is negatively correlated with perceived ease of use as specified in the Technology Acceptance Model (Davis, 1989). It can be assumed that people high in technological affinity have a clearer picture of what a robot can or cannot do. Technological orientation is connected with Robot Acceptance at Work (RAW) through two factors: on an individual level, for example, daily internet use at work, and on a national level, for example, larger mobile phone ratio (Turja & Oksanen, 2019). Affinity for Technology Interaction (Franke et al., 2019) and Need for Cognition (NFC; Cacioppo and Petty, 1982), as well as computer experience (Dijkstra et al., 1998), are moderately to strongly positive correlated. Need for Cognition is defined as the tendency of an individual to engage in and enjoy thinking (Cacioppo & Petty, 1982). Need for Cognition has a strong impact on the perception of Anthropomorphism of robots. People high in Need for Cognition tend to anthropomorphize less than people low in Need for Cognition. This is due to differences in the accessibility of egocentric information. Individuals higher in Need for Cognition more readily apply nonanthropomorphic attributions while those, lower in Need for Cognition, rather use anthropomorphic attributions (Epley et al., 2007). This results in differences, especially when attributing characteristics of Agency, Sociability, and Animacy to robots (Spatola & Wykowska, 2021). Need for Cognition and Openness are investment traits. They determine where and in which amount people invest cognitive effort over time (von Stumm, 2013). When interacting with robots, the personality trait of Openness should be considered influential. Individuals high in Openness are, for example, inquisitive about various domains of knowledge, and take an interest in unusual ideas or people (Ashton & Lee, 2009). Openness facilitates the interaction with robots in such a way that it significantly correlates with robot-led cognitive testing of elderly people (Rossi et al., 2020). This suggests that by being receptive to new ideas and experiences, the novelty of robots triggers curiosity rather than anxiety.

Possible associations between personality variables and acceptance of robots is a rather new topic. At this background, we decided to include not only Openness as a dimension of the HEXACO model (Ashton & Lee, 2009), but to investigate all dimensions (i.e., Honesty-Humility, Emotionality, Extraversion, Agreeableness, Conscientiousness, and Openness) on an exploratory basis. By using the HEXACO model (Ashton & Lee, 2009), we can include the Big Five Personality Dimensions (e.g., McCrae & Costa, 1987) and the dimension of Honesty-Humility. Honesty-Humility is associated with moral aspects. The initial implication of Openness as being curious about new things can be widened to being open and willing to emphasize with others. Empathy describes the ability to understand and react adequately to others (Paulus, 2009). Empathy includes the subfacets Perspective Taking (i.e., being able to change psychological perspective spontaneously), Fantasy (i.e., being able to empathize with fictional characters), Empathic Concern (i.e., compassion and worry for people in need), and Personal Distress (i.e., self-focused emotional reactions). Higher levels of Empathy are linked to the tendency to perceive robots as fellow social agents rather than unfeeling machines (Rossi et al., 2020; Mattiassi et al., 2021). Links between familiarity with and empathic responses to robots were proposed (Mattiassi et al., 2021). Justice Sensitivity describes how people vary in how easily they perceive injustice and how strongly they react to it. These differences are stable across time and different situations (Schmitt et al., 2009). Justice Sensitivity covers four perspectives: Victim Sensitivity, Observer Sensitivity, Beneficiary Sensitivity, and Perpetrator Sensitivity (Beierlein et al., 2014). Differences in Justice Sensitivity could also have implications for what legal competencies people associate with robots. Moral Identity is one kind of social identity people use to construct self-definition which in turn is associated with moral action (Aquino & Reed, 2002). Moral Identity covers two subscales, namely Internalization and Symbolization. The dimension of Internalization depicts the self-importance of the moral characteristics. The dimension of Symbolization depicts a general sensitivity to how the moral self is perceived in terms of their actions in the world (Aquino & Reed, 2002). We included Justice Sensitivity and Moral Identity to take into account the possibility that especially social robots, designed to be companions for users, might deserve moral consideration. The question of moral standing might therefore be answered differently for these robots (Scholtz, 2010; Coeckelbergh, 2021).

### The Present Studies: Social and Legal Perception of Embodied Digital Technologies

In two studies, we aimed at replicating and extending previous findings (Meyer & Asbrock, 2018) on the Social Perception of Embodied Digital Technologies. In Study 1, we analyzed the Social Perception of people with prostheses varying in technicality as well as robots, taking into account individual differences. In Study 2, we aimed at replicating the findings of the Study 1 for robots and widened the scope by including legal attributions to robots. We will establish our Hypotheses and Research Questions related to legal attributions in the introduction for Study 2.

Social Perception varies across the dimensions of the Stereotype Content Model for people with prostheses of differing types (Meyer & Asbrock, 2018; Peters & Wischniewski, 2019). We, therefore, hypothesized in Study 1: H1: People with physical disabilities who use low-tech prostheses are generally seen as less competent than people with physical disabilities who use bionic prostheses or able-bodied individuals. We widened the scope of the Stereotype Content Model by dividing the dimension of Warmth into Sociability and Morality, following Leach et al.’s (2007) line of argumentation. We assessed how people with physical disabilities who use low-tech prostheses, people with physical disabilities who use bionic prostheses, and able-bodied individuals were perceived in general in terms of 1) Sociability, and 2) Morality (RQ1).

We expected personality variables to be associated with the Social Perception of people with physical disabilities with different kinds of prostheses. Hence, we investigated if there is an association between personality variables and the perception of people with physical disabilities who use low-tech prostheses, people with physical disabilities who use bionic prostheses, and able-bodied individuals in terms of 1) Competence, 2) Sociability, and 3) Morality (RQ2).

Furthermore, we expanded these research questions to robots to evaluate their current perception in general. We evaluated how robots with varying levels of Anthropomorphism were perceived in terms of 1) Competence, 2) Sociability, and 3) Morality (RQ3) and whether there was an association between personality variables and the perception of robots with varying levels of Anthropomorphism in terms of 1) Competence, 2) Sociability, and 3) Morality (RQ4).

Since this is one of the first studies to approach robot perception with mostly humane attributions, we evaluated whether uniquely humane adjectives could be used to describe robots with varying levels of Anthropomorphism (RQ5).

## Study 1: Social Perception of Embodied Digital Technologies

Prior to data collection, the present study was preregistered on OSF (https://osf.io/xevkp). The procedure was evaluated and approved by the Ethics Committee. It was not considered to require further ethical approvals and hence, as uncritical concerning ethical aspects according to the criteria used by the Ethics Committee, which includes aspects of the sample of healthy adults, voluntary attendance, noninvasive measures, no deception, and appropriate physical and mental demands on the subject.

## Methods

We report how we determined our sample size, all data exclusions (if any), all manipulations, and all measures in the study (Simmons et al., 2012).

### Participants

We conducted an a-priori-power analysis with G*Power (version 3.1.9.6; Faul et al., 2007) for one-way ANOVA with fixed effects. A medium effect size of 0.25 was assumed and power set to 0.95, resulting in a sample size of *N* = 462. The sample was acquired via Prolific academic (www.prolific.co), an online survey platform (Palan and Schitter, 2018). We conducted a pilot study (*N* = 30) to assess the mean processing time. By including a manipulation check, we were able to exclude participants who did not read the instructions carefully and of whom we would expect their data to be flawed. Three participants did not meet the requirements of the manipulation checks and were therefore excluded, resulting in a final sample size of *N* = 459. We checked for outliers that were specified at having rated the social dimensions outside of ± three standard deviations from the mean and ran analyses twice: once by including and once by excluding the outliers. The results did not differ from each other, which is why for further analyses, all participants are considered. The mean age of the sample was *M* = 30.02 (*SD* = 9.77). The sample consisted of 205 female, 246 male, and eight non-binary participants and was mostly highly-educated, with 47.06% having obtained a university degree (high-school diploma: 32.90%, other degrees: 19.39%, no degree: 0.65%). Countries of residence of the participants were mainly Germany (72.77%), Austria (8.06%), and Switzerland (3.27%), with 15.90% residing in other countries.

### Measures

#### Stimulus Material

The stimulus material consisted of 11 pictures of human beings with and without low- and high-tech prostheses, and different robots. All materials can be found on osf (osf.io/xsn5a). To account for different types of disabilities and prostheses, three types of disabilities (one arm, one leg, both legs) were shown. For each disability, a low- and high-tech-type prosthesis were presented. Two able-bodied individuals, one female and one male, were shown. The pictures were chosen according to the following criteria: neutral to slightly positive facial expression, neutral clothing, neutral background. People with prostheses were exclusively male to control for the influence of female stereotypes. We presented three robots with different levels of Anthropomorphism as stimulus material: On the lowest level an industrial robot, which does not possess any human-like qualities, such as a face. On the second level, a social robot (Pepper, SoftBank Robotic Europe), which possesses a face with eyes and a mouth, and its form resembles a typical human body with head, body, and arms. The highest level of Anthropomorphism is represented by an android robot, a still image taken from the movie ROBOLOVE (Arlamovsky, 2019), which is almost indiscernible from a human being. We presented the industrial robot in a typical setting and the others in neutral settings. All pictures were presented in randomized order with instructions to rate how the participants perceive the person/robot, how they think the person/robot would act/think/react, even though this first impression might be wrong and revoked later. Twenty-five adjectives on opposing ends of a semantic differential were presented in randomized order, to be rated on a five-point Likert scale (e.g., competent-incompetent, warm-cold, artificial-natural, animated-indifferent, polite-impolite, moral-immoral, see Items). For the pictures of robots, two additional choices were given in accordance with Chita-Tegmark et al. (2021): ‘does not apply to robots in general’ and ‘does not apply to this specific robot’.

#### Items

We composed items to cover the three main dimensions Competence, Sociability, and Morality, as well as Anthropomorphism, rated on a five-point Likert scale. **Competence:** We chose four items to cover Competence (e.g., competent, able) in line with previous studies (e.g., Fiske, 2018; Fiske et al., 2002; Meyer & Asbrock, 2018), and averaged these items into a scale (McDonald’s Omega = 0.71). **Sociability:** We assessed Sociability with three subscales: Warmth (three items; e.g., warm) (Fiske, 2018; Fiske et al., 2002; Meyer & Asbrock, 2018), Animacy (three items; e.g., interactive), and Likeability (two items; e.g., friendly), the latter two subscales taken from the Godspeed Questionnaire (Bartneck et al., 2009), resulting in a total of eight items for the Sociability scale. We averaged these items into a scale (McDonald’s Omega = 0.85). **Morality:** We adapted eight attributions which people high in Moral Identity possess of the German version of the Moral Identity Questionnaire (Aquino & Reed, 2002) based on theoretical considerations, that is, intelligibility and relevance, and chose corresponding antonyms to use for the present study (e.g., ethical). We averaged these items into a scale (McDonald’s Omega = 0.78). **Anthropomorphism:** We used five items from the Godspeed Questionnaire (Bartneck et al., 2009) to assess perceived Anthropomorphism of the robots (e.g., humanlike). We averaged these items into a scale (McDonald’s Omega = 0.88). **Affinity for Technology Interaction:** We used the German version of the Affinity for Technology Interaction (ATI) Scale (Franke et al., 2019). Nine items were rated on a six-point scale (anchored at *‘not true at all’* and *‘very true’*) to indicate whether people tend to act with technological systems (e.g., *I like to try out functions of new technical systems*) and averaged into a scale (Table 1) **Need for Cognition:** We used the German short version of the Need for Cognition (NFC) scale (Bless et al., 1994), comprising of sixteen items (e.g., *I consider finding new solutions to problems a fun activity*), to assess NFC. The items were rated on a seven-point scale, anchored at *1 = strong disagreement* and *7 = strong agreement*. We calculated a sum score (Table 1). **HEXACO Personality Dimensions:** The HEXACO Personality Inventory (Ashton & Lee, 2009) consists of six scales: Honesty-Humility (H), Emotionality (E), Extraversion (X), Agreeableness (A), Conscientiousness (C), and Openness to Experience (O). For the present study, we used the 60-item version which includes 10 items for each dimension (e.g., *having a lot of money is not especially important to me (H), I sometimes can’t help worrying about little things (E), I feel reasonably satisfied with myself overall (X), I tend to be lenient in judging other people (A), I often push myself very hard when trying to achieve a goal (C), I like people who have unconventional views (O)*), rated on a five-point scale, anchored at *1 = strongly disagree* and *5 = strongly agree*. We averaged items of the corresponding scales (Table 1). **Empathy:** We used the Saarbrücker Personality Questionnaire SPF (IRI) (Paulus, 2009) for assessing empathy. The SPF is the German version of the Interpersonal Reactivity Index (IRI) and consists of four scales: Perspective Taking (PT), Fantasy (FS), Empathic Concern (EC), and Personal Distress (PD), each of which is assessed by four items (e.g., *in emergencies, I feel anxious and uncomfortable (PD), I can imagine feelings of a fictional person in a book really well (FS), I believe that every problem has two sides and try to take both into account (PT), I am touched by things even if I only observe them (EC)*). These items are rated on a five-point scale (*1 = never, 2 = seldom, 3 = sometimes, 4 = often, 5 = always)* whether this statement applies to the participant. EC, FS, and PD cover an emotional, PT a cognitive empathy factor. We averaged items of the four scales (Table 1). **Injustice Sensitivity:** We measured Injustice Sensitivity with the German short scales USS-8 (Ungerechtigkeitssensibilität-Skalen-8, Beierlein et al., 2014), which covers four perspectives (Beneficiary Sensitivity (BS), Observer Sensitivity (OS), Perpetrator Sensitivity (PS), and Victim Sensitivity (VS.)) with two items per perspective (e.g., *I feel guilty when I am better off than others for no reason (BS), I am upset when someone is undeservingly worse off than others (OS), I feel guilty when I enrich myself at the cost of others (PS)*, *It makes me angry when others are undeservingly better off than me (VS.)*)*,* which are rated on a 6-point scale anchored at *1 = not at all* and *6 = exactly*. We averaged items per perspective (Table 1). **Moral Identity:** We measured Moral Identity with the German Moral Identity Scale (Aquino & Reed, 2002), which includes two subscales, Internalization and Symbolization. Nine attributions (e.g., *honest, friendly, fair*) are presented and participants have to imagine a person with these qualities. Five items per scale are rated on a seven-point scale anchored at *1 = strongly disagree* and *7 = strongly agree* (e.g., *to be someone with these attributes is an important part of me (Internalization), I often wear clothes which identify me as someone with these attributes (Symbolization)*)*.* We averaged the items of the respective subscales (Table 1).

**TABLE 1:** Reliability analyses and descriptives of personality variables.

Scale	Subscale	Study 1 (*N* = 459)			Study 2 (*N* = 433)		
		*M*	*SD*	McDonald’s Omega	*M*	*SD*	McDonald’s Omega
ATI	-	4.25	1.01	0.93	4.15	0.96	0.92
NFC	-	14.17	15.51	0.91	12.58	14.44	0.91
HEXACO	Honesty-Humility	3.33	0.65	0.74	3.41	0.62	0.75
	Emotionality	3.13	0.69	0.81	3.12	0.62	0.79
	Extraversion	2.95	0.72	0.84	3.09	0.60	0.80
	Agreeableness	3.21	0.56	0.73	3.10	0.50	0.69
	Conscientiousness	3.56	0.59	0.77	3.65	0.52	0.75
	Openness	3.59	0.59	0.72	3.47	0.64	0.77
SPF	Empathic Concern	3.48	0.68	0.72	3.44	0.61	0.72
	Fantasy	3.44	0.72	0.72	3.39	0.69	0.75
	Personal Distress	2.73	0.77	0.74	2.74	0.77	0.81
	Perspective Taking	3.61	0.63	0.76	3.59	0.63	0.76
Injustice Sensitivity (USS-8)	Victim Sensitivity	3.73	1.28	0.78	3.63	1.30	0.86
	Observer Sensitivity	4.10	1.22	0.78	3.79	1.16	0.81
	Beneficiary Sensitivity	3.08	1.42	0.88	2.92	1.25	0.89
	Perpetrator Sensitivity	4.32	1.29	0.74	4.16	1.34	0.87
Moral Identity Scale	Internalization	5.70	0.91	0.80	5.39	0.94	0.79
	Symbolization	3.64	1.08	0.72	3.78	1.04	0.79

*Note.* ATI = Affinity for Technology Interaction, potential range = 1 to 6; NFC = Need for Cognition, potential range = -48 to +48; HEXACO, potential range = 1 to 5; SPF = Saarbrücker Personality Inventory, potential range = 1 to 5; USS-8 = Injustice Sensitivity Scales-8, potential range = 1 to 6; Moral Identity Scale, potential range = 1 to 7

### Procedure

The study was conducted as an online survey via Prolific Academic. Data were collected with Limesurvey. After giving informed consent and filling in a sociodemographic questionnaire, participants first completed the HEXACO-PI-R questionnaire (Ashton & Lee, 2009). Next, the stimulus material was presented and the participants rated each of the eleven pictures on 25 adjectives that comprised the six scales Competence, Warmth, Anthropomorphism, Animacy, Likeability, and Morality. Afterward, participants completed the five additional personality questionnaires presented in randomized order. Lastly, we asked whether the participants themselves or any of their acquaintances used prostheses. Upon finishing, participants were forwarded to Prolific Academic (http://www.prolific.co) to receive a compensation of EUR 3.60. The total processing time was approximately 30 min.

### Statistical Analysis

Each participant rated all eleven pictures. These eleven repeated measurements of the dependent variables were thus nested in participants; this was the case for Competence, ICC(1) = 0.13, *F*_(458, 1944)_ = 1.75, *p* <0 .001, ICC(2) = 0.43, Sociability, ICC(1) = 0.16, *F*_(458, 1526)_ = 1.80, *p* < 0.001, ICC(2) = 0.44, Morality, ICC(1) = 0.51, *F*_(458, 1106)_ = 4.53, *p* < 0.001, ICC(2) = 0.78. We thus employed Mixed Models to account for nested data. We used R (Version 4.1.1; R Core Team, 2021) and the Rpackages dplyr (Version 1.0.7), tidyverse (Version 1.3.1), tidyr (Version 1.1.3), forcats (Version 0.5.1) for data management, psych (Version 2.1.6), sjstats (Version 0.18.1), ggpubr (Version 0.4.0), sjplot (Version 2.8.9), lm. beta (Version 1.5–1), apaTables (Version 2.0.8), and ggplot2 (Version 3.3.5) for descriptive analyses, MuMln (Version 1.43.17), effects (Version 4.2–0), emmeans (Version 1.6.2.1), mulitlevel (Version 2.6), stats (Version 4.0.2), lme4 (Version 1.1–27.1), pbkrtest (Version 0.5.1) and lattice (Version 0.20–44) for fitting Mixed Models and subsequent post-hoc testing.

## Results

Visual inspection of the data revealed non-linear relationships between Competence, Sociability, and Morality, and Grade of Technicity, respectively. To account for the apparent break between human and robotic stimuli, we decided to split the data for all three attributions into two subgroups. The data structure for human stimuli revealed that instead of Grade of Technicity, Restored Function seemed to explain differences in attributions better. We rearranged the data from low-tech prostheses to bionic prostheses to able-bodied individuals. This was also in accordance with our Hypothesis 1 and Research Questions.

**FIGURE 1:**
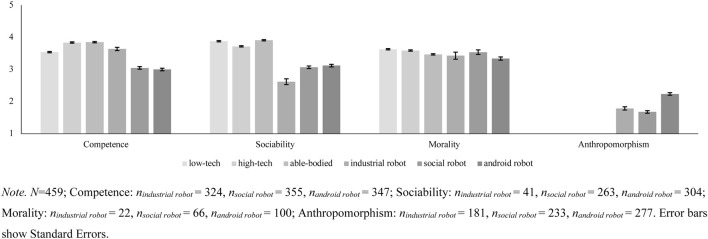
Estimated Marginal Mean Scores for Grades of Technicity and Social Perception.

For Sociability and Morality, fewer participants attributed the respective adjectives to robots (see Figure 1). We fitted three Mixed Models with random intercepts and slopes for the variables Competence, Sociability, and Morality for both subgroups (able-bodied individuals, users of low- and high-tech prostheses, and industrial, social, and anthropomorphic robots). We controlled for participants’ age, gender, and education in all models. We correlated personality dimensions of the HEXACO model, Affinity for Technology Interaction (ATI), Need for Cognition (NFC), Empathy, Injustice Sensitivity, and Moral Identity with perceptions of Competence, Sociability, and Morality for both subgroups and adjusted for multiple comparisons (Holm, 1979).

### Human Stimuli

Hypothesis 1 predicted that users of low-tech prostheses are perceived as less competent than users of bionic prostheses and able-bodied individuals. We tested the hypothesis in a Mixed Model regressing Competence on Restored Functionality (RF). A model with random intercepts and slopes fit the data best (Table 2).

**TABLE 2:** Comparison of Fit Indices for Linear Mixed Models regressing Competence on Restored Functionality for human stimuli.

Predictors	Model 0			Model 1			Model 2			Model 3		
	*b*	*SE*	*p*	*b*	*SE*	*p*	*b*	*SE*	*p*	*b*	*SE*	*p*
(Intercept)	3.83	0.02	<0.001**	3.43	0.03	<0.001**	3.43	0.03	<0.001**	4.15	0.13	<0.001**
RF				0.16	0.01	<0.001**	0.16	0.01	<0.001**	0.16	0.01	<0.001**
Gender										−0.24	0.04	<0.001**
Education										−0.05	0.02	0.003**
Age										−0.00	0.00	0.207
Random Effects												
σ^2^		0.15			0.13			0.10			0.10
*t*_00_		0.14 _id_			0.15 _id_			0.26 _id_			0.24 _id_
*t*_11_								0.03 _id.rf_			0.03 _id.rf_
ρ_01_								−0.62 _id_			-0.62 _id_
ICC		0.49			0.55			0.65			0.63
Model fit				
Marginal *R* ^*2*^		0.000			0.055			0.055			0.112
Conditional *R* ^*2*^		0.490			0.572			0.671			0.673
AIC		1927.5			1774.1			1752.0			1737.6
BIC		1943.2			1795.0			1783.4			1784.7
χ^2^					155.39**			26.09**			20.39**

*Note. N* = 459, Observations = 1,377; RF = Restored Functionality. *p < 0.05. **p < 0.01.

We found that RF was positively associated with perceptions of Competence. Post-hoc Tukey tests showed that users of low-tech prostheses were perceived as significantly less competent than users of high-tech prostheses, Δ*M* = −0.30, *SE* = 0.04, *t* = −7.59, *p* <0 .001, and as less competent than able-bodied individuals, Δ*M* = 0.31, *SE* = 0.04, *t* = 8.01, *p* <0 .001. Perceived Competence did not differ significantly between users of high-tech prostheses and able-bodied individuals, Δ*M* = 0.02, *SE* = 0.04, *t* = 0.42, *p* = 0.998. Female participants attributed significantly more Competence to individuals with and without low- and high-tech prostheses than male participants, *b* = 0.17, *SE* = 0.04, *t* = 3.79. Education was negatively associated with attributed Competence, *b* = −0.05, *SE* = 0.02, *t* = −2.96.

Research Question 1 was concerned with perceptions of users of high- and low-tech prostheses and able-bodied individuals in terms of Sociability and Morality. Perceived Sociability was not associated with Restored Functionality (Table 3).

**TABLE 3:** Comparison of Fit Indices for Linear Mixed Models regressing Sociability on Restored Functionality for the subgroup of human stimuli.

Predictors	Model 0			Model 1			Model 2		
	*b*	*SE*	*p*	*b*	*SE*	*p*	*b*	*SE*	*p*
(Intercept)	3.83	0.02	<0.001**	3.80	0.03	<0.001**	4.36	0.13	<0.001**
RF				0.02	0.01	0.100	0.02	0.01	0.100
Gender							−0.17	0.04	<0.001**
Education							−0.02	0.02	0.228
Age							−0.01	0.00	0.002**
Random Effects									
σ^2^	0.10			0.10			0.10		
*t*_00_	0.15 _id_			0.15 _id_			0.14 _id_		
ICC	0.60			0.60			0.59		
Model Fit									
Marginal *R* ^*2*^	0.000			0.001			0.044		
Conditional *R* ^*2*^	0.604			0.604			0.606		
AIC		1,475.0			1,481.6			1,481.3
BIC		1,490.7			1,502.5			1,517.9
χ^2^					0.00			6.35

*Note. N* = 459, Observations = 1,377; RF = Restored Functionality. *p < 0.05. **p < 0.01.

Perceived Morality was negatively associated with RF (Table 4). Post-hoc Tukey tests revealed that able-bodied individuals were attributed significantly less Morality than users of low-tech prostheses, Δ*M* = 0.16, *SE* = 0.02, *t* = 9.14, *p* <0 .001, and users of high-tech prostheses, Δ*M* = 0.12, *SE* = 0.02, *t* = 6.77, *p* <0 .001. Between users of high- and low-tech prostheses, the difference in perceived Morality revealed a trend toward a difference between technicity of the prostheses only on a descriptive level, Δ*M* = 0.04, *SE* = 0.02, *t* = 2.37, *p* = 0.050. Attributions of neither Sociability nor Morality were associated with the control variables. Research Question 2 is concerned with associations between personality variables and attributions of Competence, Sociability, and Morality to people with low- and high-tech prostheses and able-bodied individuals (Supplementary A).

**TABLE 4:** Comparison of Fit Indices for Linear Mixed Models regressing Morality on Restored Functionality for human stimuli.

Predictors	Model 0			Model 1			Model 2			Model 3		
	*b*	*SE*	*p*	*b*	*SE*	*p*	*b*	*SE*	*p*	*b*	*SE*	*p*
(Intercept)	3.56	0.02	<0.001**	3.72	0.03	<0.001**	3.72	0.03	<0.001	4.25	0.12	<0.001**
RF				−0.08	0.01	<0.001**	-0.08	0.01	<0.001	−0.08	0.01	<0.001**
Gender										−0.13	0.04	<0.001**
Education										−0.04	0.02	0.028*
Age										−0.00	0.00	0.015*
Random Effects												
σ^2^	0.08			0.07			0.05			0.07		
*t*_00_	0.13 _id_			0.14 _id_			0.18 _id_			0.13 _id_			
*t*_11_							0.02 _id.rf_					
ρ_01_							−0.49 _id_					
ICC	0.64			0.66			0.74			0.65		
Model Fit												
Marginal *R* ^2^	0.000			0.020			0.020			0.054		
Conditional *R* ^2^	0.639			0.669			0.741			0.670		
AIC		1,199.8			1,129.8			1,109.9			1,115.6
BIC		1,215.5			1,150.7			1,141.3			1,162.7
χ^2^					71.96**			23.92**			0.27

*Note. N* = 459, Observations = 1,377; RF = Restored Functionality. *p < 0.05. **p < 0.01.

Emotionality showed a small positive correlation with attributions of Competence (*r* = 0.25) and Sociability (*r* = 0.21). Conscientiousness showed a small positive correlation with attributed Morality (*r* = 0.17). Empathic Concern (*r*_*Competence*_ = 0.21, *r*_*Sociability*_ = 0.19, *r*_*Morality*_ = 0.18), and Internalization (*r*_*Competence*_ = 0.26, r_So*ciability*_ = 0.25, *r*_*Morality*_ = 0.21) showed small positive correlations with all three attributions of Social Perception. Attributions of Competence furthermore showed small positive correlations with Observer Sensitivity (*r* = 0.18) and Perpetrator Sensitivity (*r* = 0.20).

### Robotic Stimuli

Research Question 3 proposed that different levels of Anthropomorphism would be associated with different attributions of Competence, Sociability, and Morality. Answering this question first required establishing whether the different types of robots in the stimulus material were indeed perceived as having different levels of Anthropomorphism. We regressed Anthropomorphism on robot type with a corresponding Mixed Model. Indeed, perceptions of Anthropomorphism differed between robot types, *b* = 0.25, *SE* = 0.03, *t* = 8.44.

Post-hoc Tukey tests showed that the android robot was perceived as more anthropomorphized than the industrial robot, Δ*M* = 0.45, *SE* = 0.06, *t* = −7.81, *p* <0 .001, and the social robot, Δ*M* = 0.56, *SE* = 0.05, *t* = −10.54, *p* <0 .001. The latter two, in contrast to theoretical considerations, did not differ from each other, Δ*M* = 0.11, *SE* = 0.06, *t* = 1.82, *p* = 0.160 (Figure 1). Having established the different levels of Anthropomorphism of the stimuli, we subsequently compared perceived Competence, Sociability, and Morality across the different robot types (RQ3). Different robots were indeed associated with different levels of attributed Competence (Table 5).

**TABLE 5:** Comparison of Fit Indices for Linear Mixed Models regressing Competence on Grade of Technicity for robotic stimuli.

Predictors	Model 0			Model 1			Model 2			Model 3		
	*b*	*SE*	*p*	*b*	*SE*	*p*	*b*	*SE*	*p*	*b*	*SE*	*p*
(Intercept)	3.22	0.03	<0.001**	4.81	0.15	<0.001**	4.81	0.15	<0.001**	4.90	0.25	<0.001**
GOT				−0.32	0.03	<0.001**	−0.32	0.03	<0.001**	−0.32	0.03	<0.001**
Gender										−0.04	0.06	0.512
Education										−0.00	0.00	0.632
Age										0.00	0.03	0.901
Random Effects												
σ^2^	0.61			0.52			0.43			0.43		
*t*_00_	0.13 _id_			0.16 _id_			2.19 _id_			2.18 _id_		
*t*_11_							0.10 _id.got_			0.10 _id.got_		
ρ_01_							−0.96 _id_			−0.96 _id_		
ICC	0.18			0.23			0.36			0.37		
Model fit												
Marginal *R* ^*2*^	0.000			0.088			0.089			0.089		
Conditional *R* ^*2*^	0.180			0.298			0.421			0.423		
AIC		2,596.6			2,488.5			2,478.2			2,502.5
BIC		2,611.4			2,508.2			2,507.8			2,546.9
χ^2^					110.12**			14.26**			0.00

*Note. N* = 404, Observations = 1,026; GOT = Grade of Technicity. *p < 0.05. **p < 0.01.

Post-hoc Tukey tests showed that the industrial robot was perceived as more competent than the social robot, Δ*M* = 0.58, *SE* = 0.06, *t* = 10.54, *p* <0 .001, and as more competent than the android robot, Δ*M* = 0.64, *SE* = 0.06, *t* = 11.51, *p* <0 .001. Perceived Competence of the social and android robot did not differ, Δ*M* = 0.06, *SE* = 0.05, *t* = 1.07, *p* = 0.540. Type of robot was positively associated with perceived Sociability (Table 6).

**TABLE 6:** Comparison of Fit Indices for Linear Mixed Models regressing Sociability on Grade of. Technicity for the subgroup of robotic stimuli.

Predictors	Model 0			Model 1			Model 2		
	*b*	*SE*	*p*	*b*	*SE*	*p*	*b*	*SE*	*p*
(Intercept)	3.07	0.03	<0.001**	2.27	0.21	<0.001**	2.32	0.29	<0.001**
GOT				0.15	0.04	<0.001**	0.15	0.04	<0.001**
Gender							0.01	0.06	0.796
Education							−0.02	0.03	0.464
Age							0.00	0.00	0.745
Random Effects									
σ^2^	0.31			0.30			0.30		
*t*_00_	0.10 _id_			0.10 _id_			0.10 _id_		
ICC	0.25			0.25			0.26		
Model Fit									
Marginal *R* ^*2*^	0.000			0.020			0.021		
Conditional *R* ^*2*^	0.252			0.267			0.273		
AIC		1,172.8			1,164.4			1,188.8
BIC		1,186.1			1,182.1			1,219.7
χ^2^					10.40**			0.00

*Note. N* = 332, Observations = 608; GOT = Grade of Technicity. *p < 0.05. **p < 0.01.

Post-hoc Tukey tests showed that the industrial robot was perceived as less sociable than both the social robot, Δ*M* = -0.44, *SE* = 0.08, *t* = -5.60, *p* <0 .001, and the android robot, Δ*M* = -0.49, *SE* = 0.08, *t* = -6.35, *p* <0 .001, while the perceptions did not differ for the social robot and the android robot, Δ*M* = -0.05, *SE* = 0.04, *t* = -1.43, *p* = 0.710. For attributed Morality, we did not find evidence for an association with type of robot (Table 7). Research Question 4 was concerned with associations between personality variables and attributions of Competence, Sociability, and Mor ality to robots. Correlational analyses revealed no significant associations (Supplementary B).

**TABLE 7:** Comparison of Fit Indices for Linear Mixed Models regressing Morality on Grade of Technicity for the subgroup of robotic stimuli.

Predictors	Model 0			Model 1			Model 2		
	*b*	*SE*	*p*	*b*	*SE*	*p*	*b*	*SE*	*p*
(Intercept)	3.42	0.05	<0.001**	3.93	0.29	<0.001**	4.11	0.42	<0.001**
GOT				−0.09	0.05	0.070	−0.09	0.05	0.085
Gender							−0.12	0.09	0.160
Education							0.05	0.04	0.222
Age							−0.01	0.00	0.058
Random Effects									
σ^2^	0.20			0.20			0.20		
*t*_00_	0.11 _id_			0.11 _id_			0.10 _id_		
ICC	0.34			0.36			0.34		
Model Fit									
Marginal *R* ^*2*^	0.000			0.014			0.053		
Conditional *R* ^*2*^	0.340			0.371			0.370		
AIC		314.13			316.97			333.04
BIC		323.84			329.92			355.70
χ^2^					0.00			0.00

*Note. N* = 123, Observations = 188; GOT = Grade of Technicity. *p < 0.05. **p < 0.01.

**FIGURE 2:**
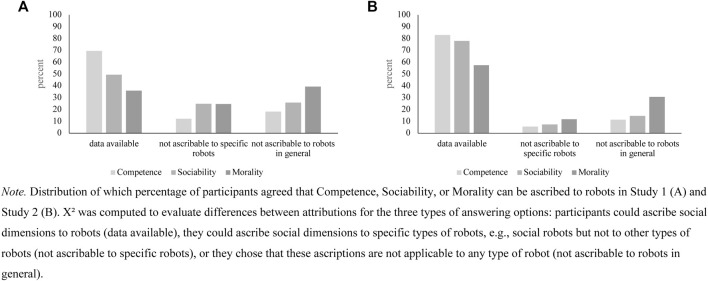
Distribution of answering options for robots for Social Perceptions.

We furthermore investigated whether uniquely humane adjectives can be used to describe robots with varying levels of Anthropomorphism (RQ5). To account for participants’ unwillingness to ascribe certain adjectives to robots, we included two possible answers: ‘does not apply to this specific robot’ and ‘does not apply to robots in general’ (Chita-Tegmark et al., 2021). Hence, we were able to further evaluate which adjectives specifically cause problems when ascribed to robots (Figure 2).

We found significant differences in the ascription of attributions to robots, χ ^*2*^ = 11.08, *df* = 2, *p* = 0.004, no differences in the willingness of attributions to certain robots, χ^*2*^ = 5.08, *df* = 2, *p* = 0.079, and significant differences in the ascription of attributions to robots in general, χ ^*2*^ = 8.19, *df* = 2, *p* = 0.017, for the three dimensions of interest. Out of 459 participants, only 22 attributed adjectives of the Morality-dimension to the industrial robot, 66 to the social robot, and 100 to the android robot. In terms of Sociability, 41 participants attributed adjectives to the industrial robot, 263 to the social robot, and 304 to the android robot. For Competence, the differences were not as large: 324 participants attributed Competence to the industrial robot, 355 to the social robot, and 347 to the android robot.

### Findings on Social Perception of Embodied Digital Technologies

Our aim was to replicate prior findings on how technicality influences the perception of people with disabilities, and to extend it to the perception of different kinds of robots. We gained insight into how non-human beings such as industrial or social robots are perceived in terms of Competence, Sociability, and Morality compared to human beings. We evaluated general attributions on the aforementioned social dimensions and investigated possible interactions of inter-individual differences on these attributions.

Users of low-tech prostheses were seen as less competent than those of high-tech prostheses. This is in line with our Hypothesis 1 (but see Meyer & Asbrock, 2018). Perceived Sociability was independent of Restored Functionality. This is surprising since ratings of Warmth, which are higher for people with disabilities, were subsumed under the umbrella of Sociability. We find this interesting since perceived Morality was higher for people with disabilities, regardless of whether they used low- or high-tech prostheses. Industrial robots were perceived as more competent than more anthropomorphized robots. Industrial robots were furthermore ascribed less Sociability than more anthropomorphized robots. Morality did not differ for types of robots. In general, individuals were reluctant to attribute inherently human characteristics such as Morality and, to some extent, Sociability to robots. We found small correlations between attributions of Competence, Sociability, and Morality and Emotionality, Empathic Concern, Perspective Taking, and Internalization for human beings. Personality variables were not correlated with attributions of the aforementioned social dimensions to robots. We will discuss those aspects together with the findings from Study 2 in the General Discussion.

## Introduction to Legal Perception of Embodied Digital Technologies

For Study 2, we chose an interdisciplinary approach. We aimed at replicating the findings for robots of Study 1. Additionally, we examined perceptions concerning legal aspects which have to be considered if robots shall become members of hybrid societies. This issue has been recognized by European institutions. They extensively discussed the question of how the EU and its member states could deal with this development. They discussed to establish an electronic person status and to define specific rights and obligations which should be granted to, or imposed on, robots. This is especially necessary because, with the growing autonomy of robots, they need to be seen less as tools than as agents (Resolution of the European Parliament, 2017/C 252/25). The connections that need to be established to identify a responsible party in case of error are often unclear and current existing concepts are no longer sufficient (Laukyte, 2019). Law in general, or legal institutions specifically, are challenged in a new way by advanced technologies (Calo, 2015). To address this development, two major dimensions of Legal Perception need to be considered: Legal Personality and subsequent Decision-Making Authority.

### Dimensions of Legal Perception: Legal Personality

Legal Personality is a mandatory condition if robots are to become part of society. The term describes legal capacity. Legal capacity is the ability to be the bearer of rights and obligations. The law takes a person’s Legal Personality and legal capacity for granted. Hence for now, Legal Personality is restricted to human actors. An extension to robots is discussed controversially. At present, robots arguably do not possess the capacities and attributions necessary to be considered as full moral or legal persons (Darling, 2012). There is a major reason why legal scholars are discussing whether robots should have rights, and why legislators need to consider this question. That is that a responsibility gap emerges when autonomous, intelligent robots act erroneously. Suppose a robot is no longer regarded as a machine or tool, which is already rejected in principle in some respects (Bryson, 2010; Bertolini, 2013), its situation can be compared to that of a substitute (Gutmann et al., 2012). In a comparable civil law constellation between human actors, the gap in responsibility of the legally represented person is bridged by the acting person. This is currently inconceivable in the case of robots. If at all, they should only be regarded as having partial legal capacity. This means that they could only be legally capable insofar as this is necessary for the applicability of the attribution rules of agency law and contractual liability for damages (Riehm, 2020). For this reason, the category of the e-person is discussed, which could be placed next to the natural and legal person. A distinction has to be made between Legal Personality and legal capacity. The e-person as a digital legal entity could participate in a legal transaction, have their assets, and be the addressee of legal obligations. Due to their legal capacity, an e-person would be able to be the bearer of rights and obligations under civil law. In addition, they would be capable of exercising fundamental rights from a constitutional perspective, and be capable of committing criminal acts (*Robotics Open Letter*, 2021). In principle, legal capacity is based on the assumption that personal status is reserved for natural persons. This raises another problem: the legal capacity of legal persons in the German legal sphere is based on the fact that a natural person is ultimately in charge (Riehm, 2020). This is different from Anglo-American law, where legal capacity is necessary for a company to sue and be sued. The addressee here is the company itself. (Kraakman et al., 2017; Watson, 2018). This is why a uniform assessment of this question poses problems when it comes to justification. There is major disagreement on the topic of robot rights. Some researchers agree that as long as a robot possesses several essential characteristics describing the ability to have rights, such as consciousness, intentionality, rationality, personality, autonomy, or sentience, they should be granted these rights (Coeckelbergh, 2010; Sparrow, 2011; Gunkel, 2018; Tavani, 2018). A contrary argument is that the granting of (fundamental) rights stands in contradiction to the Charter of Fundamental Rights in particular (*Robotics Open Letter*, 2021). Furthermore, the capacity to act in the legal sense is mandatory. This necessarily requires corporeality, but it is unclear at what level of autonomy sufficient Legal Personality is achieved (Riehm, 2020). Bryson (2010) finds very drastic words for this dilemma: “Robots should be slaves […] or servants [because] […] we design, manufacture, own, and operate robots” (*p*. 3). Bertolini (2013) argues that robots cannot be recognized as legal subjects because they are not autonomous beings. The idea that robots should have rights is therefore inconceivable (Levy, 2005).

### Dimensions of Legal Perception: Decision-Making Authority

Decision-Making Authority serves as the precondition for the capacity to act. This is defined as the ability to understand the significance and consequences of a person’s actions in the relevant context, to determine his or her will accordingly, and to act correspondingly. This capacity is presumed in the case of persons of full age. This also necessarily presupposes that this person has a Legal Personality. Decision-Making Authority includes the ability to discern between options, that is, to decide, for example, whether an action is wrong or right. It can be seen as a necessary requirement for the status of a Legal Personality.

### Legal Perception and Anthropomorphism

So far, discussions on the topic of robot rights have focused on the question of whether robots should have rights in a moral, and a next step in a legal sense. This poses questions of *who* will be granted *which* rights *under which conditions* and *how* these rights will be imposed. The question of *who* is primarily focusing on the type of robot. In many cases, literature explicitly deals with ‘social robots’ (Tavani, 2018). This category was defined as physically embodied agents which communicate and interact with humans on a social level (Darling, 2012, 2016). By employing this definition, industrial and service robots, as well as softbots (e.g., software), are excluded. Here, too, the major effect of Anthropomorphism needs to be considered. Due to physicality, perceived autonomous movement, and social behavior, these robots are viewed as potential social interaction partners. They are therefore more likely to have rights attributed to them (Darling, 2012; Turkle, 2012). Nevertheless, the legal system does not allow for this differentiated view. Whether or not robots should have rights invites discussions about necessary preconditions. Central questions are 1) *can* robots have rights, concerned with the question of capabilities of the robots, and 2) *should* robots have rights, concerned with the question of obligations towards the entity (Gunkel, 2018). By following this idea, it becomes apparent that as soon as the capabilities can be affirmed, the question of ought would also have to be answered positively. Only after theoretically affirming the attribution of any rights, the question of *how* needs to be considered. In particular, this poses the problem of how we, as human beings, can know whether a robot should be able to prove that it has the necessary characteristics to be granted rights. Gunkel (2018) describes that proof can only be granted by violating the potential rights of the robot.

To replicate the findings of Study 1 on Social Perception of robots in terms of the extended Stereotype Content Model (Fiske et al., 2002; Kervyn et al., 2015), we posed the following Research Question RQ6^1^: How are robots with different levels of Anthropomorphism are perceived in terms of 1) Competence, 2) Sociability, and 3) Morality?

We furthermore evaluated whether personality variables were associated with the attributions of 1) Competence, 2) Sociability, and 3) Morality to robots with different levels of Anthropomorphism (RQ7). Additionally, we investigated whether uniquely humane adjectives could be used to describe robots with varying levels of Anthropomorphism (RQ8).

To widen the scope to legal attributions to robots, we hypothesized that.

H2: With higher levels of Anthropomorphism, legal Decision-Making Authority is more likely ascribed to robots. H3: With higher levels of Anthropomorphism, robots are more likely to be perceived as Legal Personalities.

We furthermore evaluate d whether there is an association between the ascription of Decision-Making Authority and between the perception as a (Legal) Personality, and Social Perceptions (operationalized as Competence, Sociability, and Morality; RQ9).

### Study 2: Social and Legal Perception of Embodied Digital Technologies

Prior to data collection, the present study was preregistered on OSF (https://osf.io/xevkp). The procedure was evaluated and approved by the Ethics Committee. It was not considered to require further ethical approvals and hence, as uncritical concerning ethical aspects according to the criteria used by the Ethics Committee which includes aspects of the sample of healthy adults, voluntary attendance, noninvasive measures, no deception, and appropriate physical and mental demands on the subject.

## Methods

We report how we determined our sample size, all data exclusions (if any), all manipulations, and all measures in the study (Simmons et al., 2012).

### Participants

We conducted an a-priori-power analysis with G*Power (version 3.1.9.6; Faul et al., 2007) for a one-way ANOVA with fixed effects. A medium effect size of 0.25 was assumed and power set to 0.95, resulting in a sample size of *n* = 462. The sample was acquired via clickworker GmbH (www.clickworker.de), an online survey platform. We decided to switch to clickworker GmbH from Prolific Academic since the relevant subsample was exhausted. We conducted a pilot study (*n* = 30) to assess the mean processing time. By including a manipulation check, we were able to exclude participants who did not read the instructions carefully and of whom we would expect their data to be flawed. 29 participants did not meet the requirements of the manipulation checks and were therefore excluded, leaving the final sample size at *n* = 433. We checked for outliers that were specified at having rated the social dimensions outside of ± three standard deviations from the mean and ran analyses twice: once by including and once by excluding the outliers. The results did not differ from each other, which is why for further analyses, all participants were considered. The mean age of the sample was *M* = 39.68 (*SD* = 12.37). The sample consisted of 150 female, 280 male, and three non-binary participants. The sample was mostly highly-educated, with 44.34% having obtained a university degree (high-school diploma: 27.25%, other degrees: 28.41%). Countries of residence of the participants were Germany (92.84%), Austria (5.77%), and Switzerland (1.39%).

### Measures

#### Stimulus Material

The stimulus material consisted of three pictures of robots with varying levels of Anthropomorphism: an industrial robot, shown in a laboratory setting, a social robot (Pepper, SoftBank Robotic Europe), and an android (Arlamovsky, 2019), both of which were shown in a neutral setting. The pictures of the robots were identical to the ones used in Study 1. To assess Competence, Sociability, and Morality, all pictures were presented in random order with instructions to rate how the participants perceive the robot, how they think the robot would act/think/react, even though this first impression might be wrong and revoked later. 25 adjectives on opposing ends of a semantic differential were presented in random order, to be rated on a five-point Likert scale. Analogously to Study 1, two additional choices were given for Competence, Sociability, and Morality, in accordance with Chita-Tegmark et al. (2021): ‘does not apply to robots in general’ and ‘does not apply to this specific robot’. We presented seven items concerned with Legal Personality and twelve items concerned with Decision-Making Authority to assess Legal Perception. Participants were instructed to rate on a five-point Likert scale to which extend they agreed or disagreed with each statement (for full material see Supplementary C).

#### Items

We composed the itemset to cover the four main dimensions of Social Perception (Competence, Sociability, Morality, and Anthropomorphism, see Study 1) and two additional dimensions of Legal Perception (Decision-Making Authority and Legal Personality). Competence, Sociability, Morality, and Anthropomorphism were captured in the same way as in Study 1 (see Study 1, Items). Adjectives were presented as a semantic differential to be rated on a five-point Likert scale. We ran reliability analyses for all scales (McDonald’s Omega_Competence_ = 0.56, McDonald’s Omega_Sociability_ = 0.63, McDonald’s Omega_Morality_ = 0.67, McDonald’s Omega_Anthropomorphism_ = 0.58). We assessed personality variables by employing identical questionnaires as in Study 1 and ran subsequent reliability analyses for this sample (Table 1). **Legal Personality:** Seven items were derived from the co-authors’ legal expertise to assess the agreement of participants on whether a robot was seen as being able to hold Legal Personality (for full material see Supplementary C). We chose items that reflect facets of what would be (un-)typical for natural persons (e.g., *‘this robot is a tool’*). These items were rated on a five-point Likert scale ranging from *1 = strongly disagree, 2 = disagree, 3 = neutral, 4 = agree, 5 = strongly agree*. Due to technical issues, one item (*‘this robot is an electronic person’)* had to be excluded from all further analyses (McDonald’s Omega = 0.49).

**Decision-Making Authority:** Twelve items were derived from the co-authors’ legal expertise to evaluate whether participants would agree that robots are able to make a decision with regard to a pair of adjectives, for example, *‘this robot can distinguish between ‘white and ‘black’‘*. These items were rated on a five-point Likert scale ranging from *1 = strongly disagree, 2 = disagree, 3 = neutral, 4 = agree, 5 = strongly agree*. All evaluated terms have legal implications: To assess indeterminate legal concepts such as fault due to gross or ordinary negligence, immorality, or good faith, these abilities must be present in a person (McDonald’s Omega = 0.88).

### Procedure

The study was conducted as an online survey. Analogously to Study 1, participants first gave informed consent and filled in a sociodemographic questionnaire. Next, three pictures were presented, which were rated on a total of 25 adjectives that comprised the six scales of Social Perception. To evaluate perceived Decision-Making Authority and Legal Personality, we presented the same pictures of the robots. Participants had to decided to which extend they agreed or disagreed with the statements presented. Afterward, participants completed six questionnaires to assess personality variables. Upon finishing, participants received a code to receive a compensation of EUR 3.60. The total processing time was approximately 20 min. Additional questions concerned with the future perception of robots and their possible financial and legal responsibility were gathered for exploratory reasons and will not be reported here.

### Statistical Analysis

All participants rated all of the three pictures. These three repeated measurements of the dependent variables were thus nested in participants; this was the case for Competence (ICC(1) = 0.29, *F*_(399, 671)_ = 2.1, *p* <0 .001, ICC(2) = 0.53), Sociability (ICC(1) = 0.18, *F*_(338, 398)_ = 1.49, *p* < 0.001, ICC(2) = 0.33), Morality (ICC(1) = 0.57, *F*_(176, 192)_ = 3.72, *p* <0 .001, ICC(2) = 0.73), Anthropomorphism (ICC(1) = 0.25, *F*_(297, 316)_ = 1.68, *p* <0 .001, ICC(2) = 0.41), Decision-Making Authority (ICC(1) = 0.54, *F*_(432, 866)_ = 4.57, *p* <0 .001, ICC(2) = 0.78), and Legal Personality (ICC(1) = 0.53, *F*_(432, 866)_ = 4.42, *p* <0 .001, ICC(2) = 0.77). We thus employed mixed models to account for nested data. We used R (Version 4.1.1; R Core Team, 2021) and the Rpackages dplyr (Version 1.0.7), tidyverse (Version 1.3.1), tidyr (Version 1.1.3), forcats (Version 0.5.1) for data management, psych (Version 2.1.6), sjstats (Version 0.18.1), ggpubr (Version 0.4.0), sjplot (Version 2.8.9), lm. beta (Version 1.5–1), apaTables (Version 2.0.8), and ggplot2 (Version 3.3.5) for descriptive analyses, MuMln (Version 1.43.17), effects (Version 4.2–0), emmeans (Version 1.6.2.1), mulitlevel (Version 2.6), stats (Version 4.0.2), lme4 (Version 1.1–27.1), pbkrtest (Version 0.5.1) and lattice (Version 0.20–44) for fitting Mixed Models and subsequent post-hoc testing.

## Results

**FIGURE 3:**
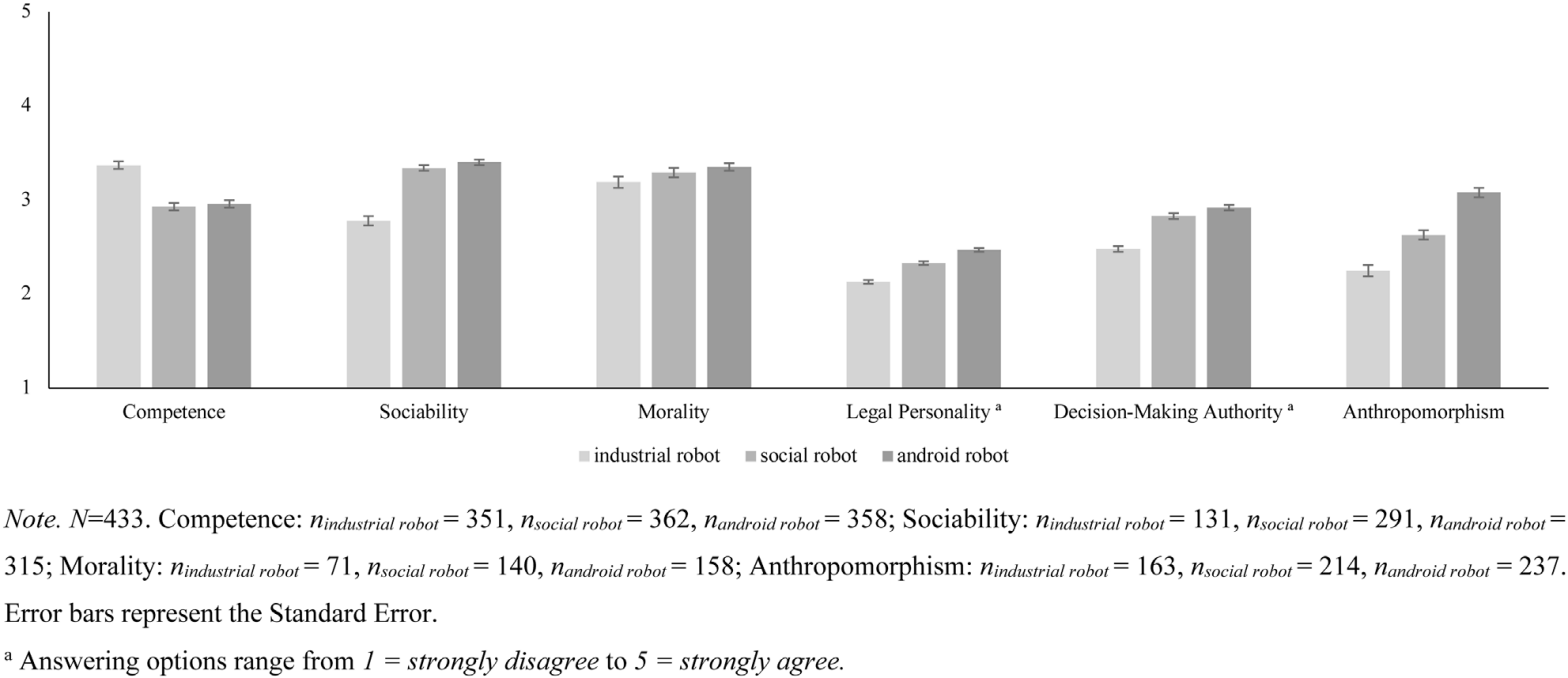
Estimated marginal mean scores for grades of technicity and social and legal perception.

We fitted three Mixed Models with random intercepts for the variables Competence, Sociability, and Morality across the different types of stimuli (Figure 3). In all models, we controlled for participants’ age, gender, and education. Personality variables, that is, personality dimensions of the HEXACO model, Need for Cognition, Affinity for Technology Interaction, Injustice Sensitivity, Moral Identity, and Empathy, with perceptions of Competence, Sociability, and Morality for both subgroups were correlated and adjusted for multiple comparisons (Holm, 1979). Furthermore, we fitted two Mixed Models with random intercepts for the variables Legal Personality and Decision-Making Authority across the three types of stimuli (Figure 3). Participants were less willing to attribute perceived Morality and Sociability to robots, independently of their anthropomorphic appearance. Competence was attributed more readily. Research Question 6 proposes that robots with different anthropomorphic appearances are associated with different perceptions of Competence, Sociability, and Morality. As for Study 1, we first evaluated Anthropomorphism to find out the three robots of the stimulus material were indeed perceived as having different levels of Anthropomorphism. We regressed Anthropomorphism on type of robot with a corresponding Mixed Model. Indeed, type of robot was positively associated with perceived Anthropomorphism, *b* = 0.41, *SE* = 0.03, *t* = 12.26. Post-hoc Tukey tests showed that, in accordance with theoretical considerations, the industrial robot was perceived as less anthropomorphic than both the social robot, Δ*M* = 0.38, *SE* = 0.07, *t* = −5.43, *p* <0 .001, and the android robot, Δ*M* = 0.82, *SE* = 0.07, *t* = −12.05, *p* <0 .001. The android robot was perceived as more anthropomorphic than the social robot, Δ*M* = 0.45, *SE* = 0.06, *t* = −7.20, *p* <0 .001 (Figure 3).

We subsequently ran three Mixed Models regressing attributions of Competence, Sociability, and Morality on type of robot.

The type of robot was associated with attributions of Competence (Table 8). Post-hoc Tukey tests showed that the industrial robot was perceived as more competent than both the social robot, Δ*M* = 0.44, *SE* = 0.05, *t* = 8.88, *p* <0 .001, and the android robot, Δ*M* = 0.41, *SE* = 0.05, *t* = 8.28, *p* <0 .001. Perceived Competence of the social robot and the android robot did not differ significantly, Δ*M* = 0.03, *SE* = 0.05, *t* = -0.58, *p* = 0.830.

**TABLE 8:** Comparison of Fit Indices for Linear Mixed Models regressing Competence on Grade of Technicity.

Predictors	Model 0			Model 1			Model 2			Model 3		
	*b*	*SE*	*p*	*b*	*SE*	*p*	*b*	*SE*	*p*	*b*	*SE*	*p*
(Intercept)	3.09	0.03	<0.001**	4.11	0.13	<0.001**	4.11	0.14	<0.001**	4.58	0.23	<0.001**
GOT				−0.20	0.03	<0.001**	−0.20	0.03	<0.001**	−0.20	0.03	<0.001**
Gender										−0.24	0.06	<0.001**
Education										−0.00	0.00	0.207
Age										0.01	0.03	0.741
Random Effects												
σ^2^	0.48			0.44			0.35			0.35		
*t*_00_	0.19 _id_			0.21 _id_			2.71 _id_			2.64 _id_		
*t*_11_							0.09 _id.got_			0.09 _id.got_		
ρ_01_							−0.96 _id_			−0.96 _id_		
ICC	0.29			0.32			0.45			0.44		
Model fit												
Marginal *R* ^*2*^	0.000			0.041			0.041			0.064		
Conditional *R* ^*2*^	0.286			0.345			0.477			0.477		
AIC		2,555.9			2,501.3			2,491.1			2,499.7
BIC		2,570.9			2,521.2			2,520.9			2,544.4
χ^2^		56.67**			14.21**			0.00

*Note. N* = 400, Observations = 1,071; GOT = Grade of Technicity. *p < 0.05. **p < 0.01.

The type of robot was furthermore positively associated with perceived Sociability (Table 9).

**TABLE 9:** Comparison of Fit Indices for Linear Mixed Models regressing Sociability on Grade of Technicity.

Predictors	Model 0			Model 1			Model 2		
	*b*	*SE*	*p*	*b*	*SE*	*p*	*b*	*SE*	*p*
(Intercept)	3.28	0.03	<0.001**	1.89	0.15	<0.001**	2.24	0.22	<0.001**
GOT				0.26	0.03	<0.001**	0.26	0.03	<0.001**
Gender							−0.11	0.05	0.034*
Education							−0.02	0.02	0.413
Age							−0.00	0.00	0.415
Random Effects									
σ^2^	0.33			0.28			0.28		
*t*_00_	0.08 _id_			0.09 _id_			0.09 _id_		
ICC	0.20			0.25			0.24		
Model Fit									
Marginal *R* ^*2*^	0.000			0.091			0.100		
Conditional *R* ^*2*^	0.202			0.314			0.320		
AIC		1,429.0			1,351.1			1,371.6
BIC		1,442.8			1,369.5			1,403.8
χ^2^					79.95**			0.00

*Note. N* = 339, Observations = 737; GOT = Grade of Technicity. *p < 0.05. **p < 0.01.

Post-hoc Tukey tests showed that the industrial robot was attributed less Sociability than the social robot, Δ*M* = 0.56, *SE* = 0.06, *t* = −10.00, *p* <0.001, and the android robot, Δ*M* = 0.63, *SE* = 0.06, *t* = −11.23, *p* <0.001. Perceived Sociability of the social and android robot did not differ significantly, Δ*M* = 0.06, *SE* = 0.04, *t* = −1.49, *p* = 0.300.

The type of robot was not associated with perceived Morality (Table 10). Research Question 7 was concerned with associations between personality variables and attributions of Competence, Sociability, and Morality to robots. Correlational analyses revealed small to moderate positive correlations between Internalization and attributions of Competence (*r* = 0.28), and Sociability (*r* = 0.38). Conscientiousness showed a moderate correlation with attributions of Competence (*r* = 0.31). Attributed Sociability showed moderate positive correlations with Affinity for Technology Interaction (*r* = 0.35), Need for Cognition (*r* = 0.33), and Honesty-Humility (*r* = 0.36) (Supplementary D).

**TABLE 10:** Comparison of Fit Indices for Linear Mixed Models regressing Morality on Grade of Technicity.

Predictors	Model 0			Model 1			Model 2		
	*b*	*SE*	*p*	*b*	*SE*	*p*	*b*	*SE*	*p*
(Intercept)	3.30	0.04	<0.001**	2.89	0.16	<0.001**	2.95	0.31	<0.001**
GOT				0.08	0.03	0.007**	0.08	0.03	0.008**
Gender							0.06	0.08	0.423
Education							−0.05	0.03	0.091
Age							0.00	0.00	0.417
Random Effects									
σ^2^	0.15			0.14			0.14		
*t*_00_	0.19 _id_			0.19 _id_			0.18 _id_		
ICC	0.57			0.57			0.56		
Model Fit									
Marginal *R* ^*2*^	0.000			0.010			0.030		
Conditional *R* ^*2*^	0.568			0.572			0.576		
AIC	571.08			571.24			590.33		
BIC	582.81			586.88			617.71		
χ^2^				1.84			0.00		

*Note. N* = 177, Observations = 369; GOT = Grade of Technicity. *p < 0.05. **p < 0.01.

Research Question 8 was concerned with evaluating whether uniquely humane adjectives can be used to describe robots with varying levels of Anthropomorphism. To account for participants’ inability to ascribe certain adjectives to robots, we included two possible answers: ‘does not apply to this specific robot’ and ‘does not apply to robots in general’ (Chita-Tegmark et al., 2021). Hence, we were able to further evaluate which adjectives specifically cause problems when ascribed to robots (Figure 2).

We found no differences in the ascription of attributions to robots, Χ^2^ = 5.00, df = 2, *p* = 0.080, no differences between the dimensions to ascribe attributions to certain robots, Χ^2^ = 2.54, df = 2, *p* = 0.280, and significant differences in the ascription of attributions to robots in general, Χ^2^ = 11.2, df = 2, *p* = 0.004, for the three dimensions of interest. Analogously to Study 1, attributions of humane adjectives, that is, terms that described Sociability or Morality, were not readily applied to robots, whereas adjectives that described Competence were attributed to robots. Out of 433 participants, only 71 attributed adjectives of the Morality-dimension to the industrial robot, 140 to the social robot, and 158 to the android robot. In terms of Sociability, 131 participants attributed adjectives to the industrial robot, 291 to the social robot, and 315 to the android robot. For Competence, the differences were not as big: 351 participants attributed Competence to the industrial robot, 362 to the social robot, and 358 to the android robot. Hypothesis 2 predicted that more Decision-Making Authority is ascribed to robots with higher-level Anthropomorphism. To test for this hypothesis, we ran a Mixed Model regressing Decision-Making Authority on the Grade of Technicity. Indeed, the type of robot was associated with Decision-Making Authority (Table 11). In general, participants were cautious to neutral to agree that robots could have Decision-Making Authority, which limitates the interpretability of the findings.

**TABLE 11:** Comparison of Fit Indices for Linear Mixed Models regressing Decision-Making Authority on Grade of Technicity.

Predictors	Model 0			Model 1			Model 2		
	*b*	*SE*	*p*	*b*	*SE*	*p*	*b*	*SE*	*p*
(Intercept)	2.74	0.03	<0.001**	1.63	0.08	<0.001**	2.00	0.21	<0.001**
GOT				0.22	0.02	<0.001**	0.22	0.02	<0.001**
Gender							−0.11	0.06	0.061
Education							−0.01	0.03	0.711
Age							−0.00	0.00	0.171
Random Effects									
σ^2^	0.26			0.21			0.21		
*t*_00_	0.30 _id_			0.32 _id_			0.32 _id_		
ICC	0.54			0.61			0.61		
Model Fit									
Marginal *R* ^*2*^	0.000			0.058			0.067		
Conditional *R* ^*2*^	0.544			0.631			0.633		
AIC	2,583.4			2,407.2			2,427.1		
BIC	2,598.9			2,427.9			2,463.2		
χ^2^				178.17**			0.00		

*Note. N* = 433, Observations = 1,299; GOT = Grade of Technicity. *p < 0.05. **p < 0.01.

Post-hoc Tukey tests revealed that the android robot was perceived as having more Decision-Making Authority than the industrial robot, Δ*M* = 0.44, *SE* = 0.03, *t* = −14.54, *p* <0 .001, and the social robot, Δ*M* = 0.09, *SE* = 0.03, *t* = −2.88, *p* = 0.010. The social robot was ascribed more Decision-Making Authority than the industrial robot, Δ*M* = 0.36, *SE* = 0.03, *t* = −11.66, *p* <0 .001.

Hypothesis 3 predicted that more anthropomorphized robots are ascribed more Legal Personality. The type of robot was associated with ascriptions of Legal Personality (Table 12). As with Decision-Making Authority, participants were reluctant to agree that robots in general could have Legal Personality.

**TABLE 12:** Comparison of Fit Indices for Linear Mixed Models regressing Legal Personality on Grade of Technicity.

Predictors	Model 0			Model 1			Model 2		
	*b*	*SE*	*p*	*b*	*SE*	*p*	*b*	*SE*	*p*
(Intercept)	2.31	0.02	<0.001**	1.47	0.06	<0.001**	1.60	0.15	<0.001**
GOT				0.17	0.01	<0.001**	0.17	0.01	<0.001**
Gender							0.03	0.04	0.528
Education							−0.01	0.02	0.436
Age							−0.00	0.00	0.145
Random Effects									
σ^2^	0.13			0.11			0.11		
*t*_00_	0.15 _id_			0.16 _id_			0.16 _id_		
ICC	0.53			0.60			0.60		
Model Fit									
Marginal *R* ^*2*^	0.000			0.065			0.070		
Conditional *R* ^*2*^	0.533			0.631			0.633		
AIC	1731.2			1,536.2			1,560.7		
BIC	1746.8			1,556.9			1,596.9		
χ^2^				196.99**			0.00		

*Note. N* = 433, Observations = 1,299; GOT, Grade of Technicity. *p < 0.05. **p < 0.01.

Post-hoc Tukey tests showed that the android robot was perceived as having more Legal Personality than the social robot, Δ*M* = 0.14, *SE* = 0.02, *t* = −6.15, *p* <0.001, and the industrial robot, Δ*M* = 0.34, *SE* = 0.02, *t* = −15.18, *p* <0.001. The social robot was ascribed more Legal Personality than the industrial robot, Δ*M* = 0.20, *SE* = 0.02, *t* = −9.04, *p* <0.001. With regard to Research Question 9, we evaluated possible associations between Social and Legal Perceptions using a correlational design with adjustment for multiple comparisons (Holm, 1979). We found strong correlations between the three social dimensions Competence and Sociability (*r* = 0.51), Competence and Morality (*r* = 0.60), and Sociability and Morality (*r* = 0.74). Decision-Making Authority showed a moderate positive correlation with Legal Personality (*r* = 0.48) and small to moderate positive correlations with Competence (*r* = 0.21), Sociability (*r* = 0.33), and Morality (*r* = 0.43).

### Findings on Social and Legal Perception of Embodied Digital Technologies

In Study 2, we investigated how robots of varying levels of Anthropomorphism are perceived in terms of social and legal dimensions. These dimensions were Competence, Sociability, Morality, Decision-Making Authority, and Legal Personality. Furthermore, we investigated whether interindividual differences would be associated with perceptions of these social dimensions. Ascriptions of Competence and Sociability were in line with the results from Study 1. The results indicated that industrial robots are seen as more competent but less sociable than more anthropomorphized robots. In contrast to Study 1, more Morality was ascribed to the android robot than to the industrial robot. This can be cautiously interpreted as that a higher level of Anthropomorphism facilitates ascriptions of uniquely humane attributions. Still, only a minority of participants were willing to ascribe adjectives of the dimension of Morality to industrial robots. Even for the most anthropomorphized robot, the android, not even half of the participants did so. Therefore, we suggest that, analogously to Study 1, attributions of Morality to robots were problematic to say at least. We found moderate positive correlations between Internalization and attributions of Competence and Sociability. Conscientiousness showed a moderate correlation with attributions of Competence. Attributed Sociability showed moderate positive correlations with Affinity for Technology Interaction, Need for Cognition, and Honesty-Humility.

Hypothesis 2 predicted that more anthropomorphized robots are attributed more Decision-Making Authority. This was supported by our data with the limitation that participants were reluctant to attribute Decision-Making Authority to robots in general. The more anthropomorphized robots, that is, the social robot and android robot, were attributed more Decision-Making Authority than the industrial robot. Hypothesis 3 predicted that more anthropomorphized robots are attributed more Legal Personality. Our results supported Hypothesis 3 with the same restriction: participants were reluctant to attribute Legal Personality to robots in general.

Legal Personality was not associated with Social Perceptions, but Decision-Making Authority was: Perceptions of Competence, Sociability, or Morality were associated with Decision-Making Authority. This can be interpreted in such a way that both constructs need to be considered in close proximity and might stem from a common background.

## General Discussion

We conducted two studies, aiming at a more thorough understanding of how individuals with disabilities using low- and high-tech prostheses, and robots are perceived in terms of social and legal dimensions. In Study 1, we aimed at replicating prior findings of changes in Social Perception if bionic prostheses are used by people with physical disabilities (Meyer & Asbrock, 2018). Furthermore, we widened the scope in two directions. We divided the social dimension of Warmth into Sociability and Morality, and we extended the focus to robots of varying levels of Anthropomorphism. Attributions of Social Perception to robots showed mixed results. Participants attributed Competence and, partly, Sociability, to robots. Conversely, participants were not willing to attribute Morality to robots, independently of the robots’ anthropomorphic level.

Study 2 aimed at replicating the findings of Study 1 for robots and extended the perception by a legal component. We could mostly replicate the findings of Study 1. Legal Perception, that is, Legal Personality and Decision-Making Authority, were partly associated with anthropomorphic appearance.

### Social Perception of Human Beings With Prostheses

We hypothesized that users of bionic prostheses would be attributed more perceived Competence than users of low-tech prostheses. Furthermore, we investigated whether there are associations between the technicity of prostheses and attributions of Social Perception, that is, Sociability and Morality.

Restored Functionality was a better predictor of perceptions of Competence, Sociability, and Morality than Grade of Technicity. Users of bionic prostheses were perceived as more competent than users of low-tech prostheses. At the same time, they were ascribed more Morality than able-bodied individuals. This indicates that their disability still affected Social Perception (Meyer & Asbrock, 2018). Perception of Sociability was not associated with types of prostheses. This lends weight to the approach of the division of the Stereotype Content Model dimension Warmth into Morality and Sociability (Leach et al., 2007; Heflick et al., 2011; Kervyn et al., 2015). Apparently, perceptions between these two dimensions differ, and by pooling them, explanatory value might be lost or at least reduced.

### Social Perception of Robots

We investigated whether attributions of Competence, Sociability, and Morality were associated with levels of Anthropomorphism of robots. Furthermore, we investigated whether people were willing to ascribe these attributions to robots. The findings were predominantly in line with prior findings. The results indicated that effects of Anthropomorphism on Social Perceptions are present. The general public is still cautious of granting robots attributions in previously uniquely humane domains such as Sociability and Morality. This might be partly explained by the fact that the majority of people are not in direct contact with robots. Therefore, they might not be able or willing to assess robots in an unbiased way. As Naneva et al. (2020) pointed out, there is evidence that attitudes towards robots or cyborgs are currently based on fiction and threatening images (e.g., The Terminator; Cameron, 1984) rather than facts or objective reality, respectively. This assumption is also strengthened by recent research based on intergroup relations. Sarda Gou et al., 2021) showed that direct contact with robots positively affected participants’ explicit and implicit attitudes toward robots. Direct contact might also be the crucial factor in why we found rather reserved attributions to robots while people working with robots attribute positive and negative human characteristics to them (Sauppé & Mutlu, 2015). Further studies should address whether differences in perception persist if people work with robots or not and whether perceptions in work settings can be conferred to social settings. We assume that attitudes towards and emotions evoked by robots will become more realistic and objective in the long run. Longitudinal studies should be conducted to assess and monitor those changes. In comparing Study 1 and Study 2, it is notable that the percentage of individuals who ascribed social attributions to robots changed. Fewer people ascribed moral or sociable capacities to robots if they were shown in close succession with human beings rather than when the stimuli only contained robots. We suggest that this might be due to robots being perceived as *the other* (Gunkel, 2018), and therefore making ascriptions of social dimensions harder.

### The Role of Individual Differences in Social Perception

Associations between personality variables and Social Perceptions remain inconclusive. In Study 1, we found evidence that people with higher scores in Emotionality, Empathic Concern, Internalization and, in parts, Observer Sensitivity and Perpetrator Sensitivity, tended to attribute more Competence, Sociability, and Morality. This was independent from whether people wore prostheses of any type or not. Empathic Concern, as well as Observer Sensitivity and Perpetrator Sensitivity are moderately correlated, as is Emotionality with these three variables. This might point to a specific disposition of people being especially compassionate and empathetic. They might tend to ascribe more positive attributions to other people. Internalization, the degree to which a persons’ private views are centered on moral traits (Aquino & Reed, 2002), might be interpreted in a similar direction. For robots, associations between personality variables and attributions of social dimensions differed between Study 1 and 2. In Study 1, no associations were found. We consider that by mixing human and robotic stimuli, we made the differences between the two subgroups more salient. Attributions were, therefore, more conservative. This was reflected in the low number of participants who decided to attribute Sociability or Morality to robots. In Study 2, only robotic stimuli were presented. Slightly more participants attributed Competence, Sociability, and Morality to robots, even though we still interpret these results carefully. Higher Internalization, as was true for the human subgroup of Study 1, was associated with higher overall attributions of Competence and Sociability. This could be interpreted in such a way that this personality trait is important for any attribution really, independently from *who* or *what* it is attributed to. Attributions of Sociability were correlated with Affinity for Technology Interaction and Need for Cognition. Higher positive interest in technical gadgets and subsequently in the interaction with robots (de Graaf & Ben Allouch, 2013) leads to more attributions of human adjectives to robots. Furthermore, Need for Cognition is associated with the ability to access nonanthropomorphic representations more readily instead of relying on Anthropomorphism (Epley et al., 2007). To conclude, we found evidence that personality variables are associated with Social Perception, but, especially for the area of robots, further research is needed.

### Legal Perception of Robots

Currently, discussion about granting robot rights, and the basis necessary for it, is conducted by experts from various fields, legislative institutions, and their advisors. Therefore, we investigated whether the participants associate typical legal attributions with the stimuli. We focused on two concepts: Legal Personality and Decision-Making Authority. We selected only these two legal concepts as they relate to natural persons. In the future, other categories may be added, such as the issue of tort capacity. To what extent robots may have human characteristics and to what extent they might also have legal capacity is currently the subject of a multi-layered debate. The discussion on rights for robots is focused on social robots, as by Tavani (2018) or Darling (2012, 2016). They only deal with the category of robots that communicate and interact on the social level. This definition of social robots as physically embodied agents that communicate and interact with humans on a social level (Darling, 2012, 2016) excludes industrial and service robots as well as softbots (e.g., software) from the grant of rights. The results of our study can be cautiously interpreted as partly supporting this assumption. Overall, participants were neutral to dismissive to agree with the statements which indicated Decision-Making Authority for robots. Within this range, more anthropomorphic robots were attributed more Decision-Making Authority. Similar to what was described by Darling (2012) and Tavani (2018), there is a tendency that the more human-like a robot looks and is perceivably programmed, the more they are seen as capable of making legally relevant decisions corresponding to humans. Therefore, they are seen as proficient in this respect. This might be caused by the fact that we, as humans, project our characteristics onto other human-like beings. Therefore, we feel more comfortable with granting them a certain legal standing. Nonetheless, further research is needed. If such a result is indeed confirmed on the long run, it would have meaningful implications for the use of robots. If the ascription of Decision-Making Authority is not in focus, anthropomorphic appearance is not necessary. But if this authority is of importance (e.g., in advertising), then the appearance should be considered. The same is true for the ascription of Legal Personality to robots. Participants were neutral to dismissive to agree that robots could possess Legal Personality. Within this range, more anthropomorphized robots were perceived as more eligible for Legal Personality.

Previous research agrees that once a robot has certain inherent abilities that are human-like, especially if they have some sort of consciousness, they should also be granted rights (Sparrow, 2004, 2011; Singer and Sagan, 2009; Coeckelbergh, 2010; Gunkel, 2018; Tavani, 2018). While Bryson, 2010) considers robots as tools, or the basic legal capacity is denied (*Robotics Open Letter*, 2021), a trend can be observed that this is confirmed with regard to industrial, but less so for social and android robots. From this, it can be deduced that in a further legal discussion about robot rights, the similarity to humans must be included. This has to be done at a more sophisticated level than has been the case to date to increase the acceptance of new regulations. Keeping the aforementioned limitation in mind, these results indicate that there might be an association between levels of Anthropomorphism and acceptance of robots as members of a hybrid society in a legal sense. This applies to both appearance and the internal possibility of making decisions that ultimately have a legal effect. These aspects should be taken into account in the justification of new interpretations of norms.

## Limitations

The present studies are not without limitations. For one, we evaluated the Social Perception of robots and individuals with and without physical disabilities with low- and high-tech prostheses in a relatively straightforward way by presenting unmoving pictures. Research has shown that the existence of movement plays a role in how robots are perceived (Kupferberg et al., 2011). This is why we see the present research as the first step towards a better understanding of the Social Perception of robots. We will take into the field as a subsequent next step. Pictures of individuals are highly influenced by personal taste. We decided on presenting pictures of actual human beings, so they differed in their physical appearance and might have influenced their perception. We refrained from using the RoSA Scale (Carpinella et al., 2017) to measure the Social Perception of robots and instead used scales from the Godspeed Inventory (Bartneck et al., 2009) and the Stereotype Content Model (Fiske, 2018; Meyer & Asbrock, 2018), since we not only investigated perceptions of robots but also of human beings. This decision comes with certain downfalls. The subscales Animacy and Anthropomorphism of the Godspeed Inventory (Bartneck et al., 2009) were shown to load on the same factor. Nevertheless, the core dimensions of Social Perception are unaffected by this decision (Scheunemann et al., 2020). Since we were dipping into a new field of research with employing legal questions in a survey setting, scales will need revision to provide a more conclusive insight. One should therefore interpret the results on Legal Perception of robots cautiously. Furthermore, technical issues accounted for the loss of one item of the scale for Legal Personality, which had a detrimental effect on the interpretability.

## Conclusions

The present study shows that perceptions of Competence and Morality of users of prostheses varies as a function of technical sophistication of the prostheses. Conversely, we did not find any differences in perceived Sociability. The Social Perception of robots is strongly dependent on the perceived Anthropomorphism of the specific robot. This is not to say that robots can be easily ascribed humane attributions. Some attributes, for example, in terms of Competence or Sociability, can be used to describe both humans and robots. For more abstract terms like moral perceptions, a difference is made between humans and robots. In general, the public is reluctant to see robots as personalities in a psychological and legal sense. Therefore, we infer that at the present time, robots are not perceived as equivalents to ‘co-workers’. This might change with greater availability of anthropomorphized robots. To sum it up, despite the downfalls, we can present new insights into a field which, in the future, will be of great importance for researchers and society alike.

## Data Availability

The original contributions presented in the study are included in the article/Supplementary Materials, further inquiries can be directed to the corresponding author.

## Footnotes

1 We decided to consecutively number Research Questions and Hypotheses for Study 1 and Study 2.
