# Supplementary material for: Embodied Digital Technologies: First Insights in the Social and Legal Perception of Robots and Users of Prostheses
Source: Front Robot AI. 2022 Apr 11;9:787970. doi: 10.3389/frobt.2022.787970 (PMC9037747; doi:10.3389/frobt.2022.787970)
Supplement: Supplementary file 1 [file DataSheet1.docx]

**Supplement A**

*Means, Standard Deviations, and Correlations between attributions of Competence, Sociability, and Morality and personality variables for human stimuli*

| 21 |  |  |  |  |  |  |  |  |  |  |  |  |  |  |  |  |  |  |  |  |  |
| --- | --- | --- | --- | --- | --- | --- | --- | --- | --- | --- | --- | --- | --- | --- | --- | --- | --- | --- | --- | --- | --- |
| 20 |  |  |  |  |  |  |  |  |  |  |  |  |  |  |  |  |  |  |  |  | .32** |
| 19 |  |  |  |  |  |  |  |  |  |  |  |  |  |  |  |  |  |  |  | .33** | .12 |
| 18 |  |  |  |  |  |  |  |  |  |  |  |  |  |  |  |  |  |  | .47** | .22** | .12 |
| 17 |  |  |  |  |  |  |  |  |  |  |  |  |  |  |  |  |  | .50** | .36** | .25** | .09 |
| 16 |  |  |  |  |  |  |  |  |  |  |  |  |  |  |  |  | .37** | .21** | -.02 | .01 | .03 |
| 15 |  |  |  |  |  |  |  |  |  |  |  |  |  |  |  | -.12 | .08 | .12 | .15 | .21** | .15 |
| 14 |  |  |  |  |  |  |  |  |  |  |  |  |  |  | -.07 | .28** | .24** | .29** | .19* | .05 | -.04 |
| 13 |  |  |  |  |  |  |  |  |  |  |  |  |  | .16 | .29** | .05 | .12 | .14 | .08 | .23** | .25** |
| 12 |  |  |  |  |  |  |  |  |  |  |  |  | .41** | .27** | .34** | -.03 | .43** | .41** | .44** | .45** | .26** |
| 11 |  |  |  |  |  |  |  |  |  |  |  | .27** | .31** | .02 | .25** | -.02 | .23** | .19** | .22** | .19* | .14 |
| 10 |  |  |  |  |  |  |  |  |  |  | .05 | .07 | .01 | -.21** | .11 | -.01 | .03 | -.09 | .09 | .18* | .16 |
| 9 |  |  |  |  |  |  |  |  |  | -.03 | -.03 | .12 | .10 | -.17* | .31** | -.33** | -.09 | .03 | .09 | .11 | .08 |
| 8 |  |  |  |  |  |  |  |  | .06 | .22** | .05 | .07 | .05 | -.47** | .11 | -.21** | -.05 | -.12 | -.02 | .10 | .31** |
| 7 |  |  |  |  |  |  |  | -.19** | -.11 | -.02 | .15 | .49** | .32** | .62** | .07 | .21** | .37** | .37** | .29** | .22** | .11 |
| 6 |  |  |  |  |  |  | .10 | .06 | .20** | .20** | .15 | .29** | .02 | -.03 | .12 | -.26** | .14 | .21** | .48** | .17 | .00 |
| 5 |  |  |  |  |  | .11 | -.13 | .30** | -.05 | .33** | .37** | .17 | .15 | -.29** | .28** | -.12 | .06 | .03 | .09 | .14 | .10 |
| 4 |  |  |  |  | .40** | -.10 | -.21** | .07 | .08 | .02 | .03 | -.04 | -.05 | -.16 | .10 | -.11 | -.06 | -.09 | -.16 | -.02 | -.06 |
| 3 |  |  |  | .13 | .07 | .06 | .16 | .04 | .11 | .17* | .02 | .18* | .14 | .05 | .13 | .03 | .07 | .05 | .11 | .21** | .16 |
| 2 |  |  | .84** | .10 | .12 | .09 | .21** | .03 | .07 | .13 | .07 | .19* | .15 | .07 | .12 | -.01 | .10 | .09 | .13 | .25** | .13 |
| 1 |  | .78** | .74** | .05 | .07 | .12 | .25** | -.04 | .04 | .07 | .15 | .21** | .16 | .15 | .10 | .05 | .18** | .14 | .20** | .26** | .08 |
| SD | 0.44 | 0.42 | 0.40 | 1.01 | 15.51 | 0.65 | 0.69 | 0.72 | 0.56 | 0.59 | 0.59 | 0.68 | 0.72 | 0.77 | 0.63 | 1.28 | 1.22 | 1.42 | 1.29 | 0.91 | 1.08 |
| M | 3.74 | 3.83 | 3.56 | 4.25 | 14.17 | 3.33 | 3.13 | 2.95 | 3.21 | 3.56 | 3.59 | 3.48 | 3.44 | 2.73 | 3.61 | 3.73 | 4.10 | 3.08 | 4.32 | 5.70 | 3.64 |
| Variable | 1 Competence | 2 Sociability | 3 Morality | 4 ATI | 5 NFC | 6 Honesty-Humility | 7 Emotionality | 8 Extraversion | 9 Agreeableness | 10 Conscientiousness | 11 Openness | 12 Empathic Concern | 13 Fantasy | 14 Personal Distress | 15 Perspective Taking | 16 Victim Sensitivity | 17 Observer Sensitivity | 18 Beneficiary Sensitivity | 19 Perpetrator Sensitivity | 20 Internalization | 21 Symbolization |

*Note. N* = 459. ATI = Affinity for Technology Interaction; NFC = Need for Cognition. **p* < .05, Holm-corrected for multiple testing. ***p* < .01 Holm-corrected for multiple testing.

**Supplement B**

*Means, Standard Deviations, and Correlations between attributions of Competence, Sociability, and Morality and personality variables for robotic stimuli*

| 21 |  |  |  |  |  |  |  |  |  |  |  |  |  |  |  |  |  |  |  |  |  |
| --- | --- | --- | --- | --- | --- | --- | --- | --- | --- | --- | --- | --- | --- | --- | --- | --- | --- | --- | --- | --- | --- |
| 20 |  |  |  |  |  |  |  |  |  |  |  |  |  |  |  |  |  |  |  |  | .32** |
| 19 |  |  |  |  |  |  |  |  |  |  |  |  |  |  |  |  |  |  |  | .33** | .12 |
| 18 |  |  |  |  |  |  |  |  |  |  |  |  |  |  |  |  |  |  | .47** | .22** | .12 |
| 17 |  |  |  |  |  |  |  |  |  |  |  |  |  |  |  |  |  | .50** | .36** | .25** | .09 |
| 16 |  |  |  |  |  |  |  |  |  |  |  |  |  |  |  |  | .37** | .21** | -.02 | .01 | .03 |
| 15 |  |  |  |  |  |  |  |  |  |  |  |  |  |  |  | -.12 | .08 | .12 | .15 | .21** | .15 |
| 14 |  |  |  |  |  |  |  |  |  |  |  |  |  |  | -.07 | .28** | .24** | .29** | .19* | .05 | -.04 |
| 13 |  |  |  |  |  |  |  |  |  |  |  |  |  | .16 | .29** | .05 | .12 | .14 | .08 | .23** | .25** |
| 12 |  |  |  |  |  |  |  |  |  |  |  |  | .41** | .27** | .34** | -.03 | .43** | .41** | .44** | .45** | .26** |
| 11 |  |  |  |  |  |  |  |  |  |  |  | .27** | .31** | .02 | .25** | -.02 | .23** | .19** | .22** | .19* | .14 |
| 10 |  |  |  |  |  |  |  |  |  |  | .05 | .07 | .01 | -.21** | .11 | -.01 | .03 | -.09 | .09 | .18* | .16 |
| 9 |  |  |  |  |  |  |  |  |  | -.03 | -.03 | .12 | .10 | -.17 | .31** | -.33** | -.09 | .03 | .09 | .11 | .08 |
| 8 |  |  |  |  |  |  |  |  | .06 | .22** | .05 | .07 | .05 | -.47** | .11 | -.21** | -.05 | -.12 | -.02 | .10 | .31** |
| 7 |  |  |  |  |  |  |  | -.19* | -.11 | -.02 | .15 | .49** | .32** | .62** | .07 | .21** | .37** | .37** | .29** | .22** | .11 |
| 6 |  |  |  |  |  |  | .10 | .06 | .20** | .20** | .15 | .29** | .02 | -.03 | .12 | -.26** | .14 | .21** | .48** | .17 | .00 |
| 5 |  |  |  |  |  | .11 | -.13 | .30** | -.05 | .33** | .37** | .17 | .15 | -.29** | .28** | -.12 | .06 | .03 | .09 | .14 | .10 |
| 4 |  |  |  |  | .40** | -.10 | -.21** | .07 | .08 | .02 | .03 | -.04 | -.05 | -.16 | .10 | -.11 | -.06 | -.09 | -.16 | -.02 | -.06 |
| 3 |  |  |  | .09 | .00 | -.36 | -.28 | .45 | -.23 | -.16 | -.28 | -.32 | -.34 | -.46 | -.34 | .33 | -.19 | -.19 | -.17 | .02 | .16 |
| 2 |  |  | .57 | -.09 | .27 | -.09 | .04 | .06 | -.29 | .12 | .26 | .02 | -.08 | .27 | -.04 | .24 | -.03 | .29 | .02 | .02 | .36 |
| 1 |  | -.13 | .67 | -.02 | .01 | -.05 | .05 | .02 | .06 | .03 | -.05 | .11 | .11 | -.04 | .02 | .06 | .06 | .02 | -.02 | .14 | .08 |
| SD | 0.57 | 0.35 | 0.45 | 1.01 | 15.51 | 0.65 | 0.69 | 0.72 | 0.56 | 0.59 | 0.59 | 0.68 | 0.72 | 0.77 | 0.63 | 1.28 | 1.22 | 1.42 | 1.29 | 0.91 | 1.08 |
| M | 3.23 | 2.84 | 3.33 | 4.25 | 14.17 | 3.33 | 3.13 | 2.95 | 3.21 | 3.56 | 3.59 | 3.48 | 3.44 | 2.73 | 3.61 | 3.73 | 4.10 | 3.08 | 4.32 | 5.70 | 3.64 |
| *N* | 266 | 35 | 12 | 459 | 459 | 459 | 459 | 459 | 459 | 459 | 459 | 459 | 459 | 459 | 459 | 459 | 459 | 459 | 459 | 459 | 459 |
| Variable | 1 Competence | 2 Sociability | 3 Morality | 4 ATI | 5 NFC | 6 Honesty-Humility | 7 Emotionality | 8 Extraversion | 9 Agreeableness | 10 Conscientiousness | 11 Openness | 12 Empathic Concern | 13 Fantasy | 14 Personal Distress | 15 Perspective Taking | 16 Victim Sensitivity | 17 Observer Sensitivity | 18 Beneficiary Sensitivity | 19 Perpetrator Sensitivity | 20 Internatlization | 21 Symbolization |

*Note.* ATI = Affinity for Technology Interaction; NFC = Need for Cognition. **p* < .05, Holm-corrected for multiple comparisons. ***p* < .01, Holm-corrected for multiple comparisons.

**Supplement C**

**Legal Perception**

The items were derived from the co-author’s legal expertise to assess the agreement of participants on whether robots are able to make decisions, and whether robots were seen as being able to hold Legal Personality. The items were presented as a semantic differential to be rated on a five-point Likert scale.

**Decision Making Authority**

This robot can distinguish between "right" and "wrong".

This robot can distinguish between "careful" and "careless".

This robot can distinguish between "white" and "black".

This robot can distinguish between "important" and "unimportant".

This robot can distinguish between "good-natured" and "hostile".

This robot can distinguish between "fast" and "slow".

This robot can distinguish between "own" and "foreign".

This robot can distinguish between "near" and "far".

This robot can distinguish between "conscious" and "unconscious".

This robot can distinguish between "guilty" and "innocent".

This robot can distinguish between "immediate" and "long-term".

This robot can distinguish between " capable of insight " and " not capable of insight ".

**Legal Personality**

This robot is a tool.

This robot is an electronic person.

This robot obeys instructions.

This robot can make independent decisions.

This robot can legally bind itself.

I believe that this robot cannot make a mistake.

I believe that the legal regulations are explicit when the robot makes a mistake.

**Supplement D**

*Means, Standard Deviations, and Correlations between attributions of Competence, Sociability, and Morality and personality variables*

| 21 |  |  |  |  |  |  |  |  |  |  |  |  |  |  |  |  |  |  |  |  |  |
| --- | --- | --- | --- | --- | --- | --- | --- | --- | --- | --- | --- | --- | --- | --- | --- | --- | --- | --- | --- | --- | --- |
| 20 |  |  |  |  |  |  |  |  |  |  |  |  |  |  |  |  |  |  |  |  | .20** |
| 19 |  |  |  |  |  |  |  |  |  |  |  |  |  |  |  |  |  |  |  | .31** | .02 |
| 18 |  |  |  |  |  |  |  |  |  |  |  |  |  |  |  |  |  |  | .45** | .06 | .14 |
| 17 |  |  |  |  |  |  |  |  |  |  |  |  |  |  |  |  |  | .51** | .36** | .11 | .07 |
| 16 |  |  |  |  |  |  |  |  |  |  |  |  |  |  |  |  | .54** | .34** | .17 | .00 | .05 |
| 15 |  |  |  |  |  |  |  |  |  |  |  |  |  |  |  | -.09 | .11 | .13 | .22** | .30** | .18* |
| 14 |  |  |  |  |  |  |  |  |  |  |  |  |  |  | .00 | .25** | .29** | .29** | .11 | .07 | .13 |
| 13 |  |  |  |  |  |  |  |  |  |  |  |  |  | .26** | .27** | .19* | .27** | .22** | .17 | .30** | .17 |
| 12 |  |  |  |  |  |  |  |  |  |  |  |  | .52** | .37** | .37** | .10 | .36** | .31** | .22** | .40** | .25** |
| 11 |  |  |  |  |  |  |  |  |  |  |  | .22** | .28** | -.03 | .25** | -.02 | .11 | .05 | .09 | .20** | .09 |
| 10 |  |  |  |  |  |  |  |  |  |  | .20** | .09 | .14 | -.19* | .21** | -.11 | -.03 | -.06 | .10 | .26** | .06 |
| 9 |  |  |  |  |  |  |  |  |  | -.01 | -.02 | .13 | .02 | -.20** | .36** | -.37** | -.19* | .00 | .10 | .10 | .08 |
| 8 |  |  |  |  |  |  |  |  | .19* | .09 | .04 | -.02 | -.01 | -.45** | .06 | -.21** | -.17* | -.28** | -.19* | -.04 | .16 |
| 7 |  |  |  |  |  |  |  | -.39** | -.18* | -.02 | .06 | .47** | .36** | .65** | .04 | .27** | .37** | .29** | .22** | .23** | .07 |
| 6 |  |  |  |  |  |  | -.09 | .07 | .34** | .18* | .15 | .08 | -.04 | -.17 | .26** | -.33** | -.04 | -.01 | .37** | .24** | -.07 |
| 5 |  |  |  |  |  | .21** | -.15 | .25** | -.01 | .50** | .42** | .01 | .08 | -.29** | .17* | -.16 | -.03 | -.10 | .07 | .19* | .00 |
| 4 |  |  |  |  | .40** | .10 | -.22** | .22** | .12 | .21** | .10 | -.05 | .09 | -.28** | .11 | -.04 | -.02 | -.01 | -.04 | .08 | .06 |
| 3 |  |  |  | .36 | .36 | .28 | -.17 | .13 | .02 | .34 | .22 | .41 | .12 | -.18 | .16 | -.04 | .02 | -.10 | -.21 | .41 | .22 |
| 2 |  |  | .74** | .35* | .33* | .36* | -.12 | .09 | .18 | .30 | .05 | .25 | .05 | -.21 | .26 | -.04 | .07 | -.09 | .08 | .38** | .08 |
| 1 |  | .51** | .60** | .12 | .20 | .14 | .03 | .07 | .06 | .31** | .07 | .19 | .19 | -.01 | .15 | -.06 | .04 | -.11 | .04 | .28** | .01 |
| SD | 0.59 | 0.44 | 0.47 | 0.96 | 14.44 | 0.62 | 0.62 | 0.60 | 0.50 | 0.52 | 0.64 | 0.61 | 0.69 | 0.77 | 0.63 | 1.30 | 1.16 | 1.25 | 1.34 | 0.94 | 1.04 |
| M | 3.10 | 3.14 | 3.20 | 4.15 | 12.58 | 3.41 | 3.12 | 3.09 | 3.10 | 3.65 | 3.47 | 3.44 | 3.39 | 2.74 | 3.59 | 3.63 | 3.79 | 2.92 | 4.16 | 5.39 | 3.78 |
| *N* | 303 | 117 | 62 | 433 | 433 | 433 | 433 | 433 | 433 | 433 | 433 | 433 | 433 | 433 | 433 | 433 | 433 | 433 | 433 | 433 | 433 |
| Variable | 1 Competence | 2 Sociability | 3 Morality | 4 ATI | 5 NFC | 6 Honesty-Humility | 7 Emotionality | 8 Extraversion | 9 Agreeableness | 10 Conscientiousness | 11 Openness | 12 Empathic Concern | 13 Fantasy | 14 Personal Distress | 15 Perspective Taking | 16 Victim Sensitivity | 17 Observer Sensitivity | 18 Beneficiary Sensitivity | 19 Perpetrator Sensitivity | 20 Internalization | 21 Symbolization |

*Note.* ATI = Affinity for Technology Interaction; NFC = Need for Cognition. **p* < .05 Holm-corrected for multiple comparisons. ***p* < .01 Holm-corrected for multiple comparisons.
